# Polyphenols in Dental Applications

**DOI:** 10.3390/bioengineering7030072

**Published:** 2020-07-07

**Authors:** Naji Kharouf, Youssef Haikel, Vincent Ball

**Affiliations:** 1 Faculté de Chirurgie Dentaire, Université de Strasbourg, 8 rue Sainte Elisabeth, 67000 Strasbourg, France; dentistenajikharouf@gmail.fr (N.K.); youssef.haikel@unistra.fr (Y.H.); 2 Institut National de la Santé et de la Recherche Médicale, Unité Mixte de Recherche 1121, 11 rue Humann, 67085 Strasbourg, France

**Keywords:** polyphenols, interactions with collagen, dentin, enamel, dental resins, antibacterial activity

## Abstract

(1) Background: polyphenols are a broad class of molecules extracted from plants and have a large repertoire of biological activities. Biomimetic inspiration from the effects of tea or red wine on the surface of cups or glass lead to the emergence of versatile surface chemistry with polyphenols. Owing to their hydrogen bonding abilities, coordination chemistry with metallic cations and redox properties, polyphenols are able to interact, covalently or not, with a large repertoire of chemical moieties, and can hence be used to modify the surface chemistry of almost all classes of materials. (2) Methods: the use of polyphenols to modify the surface properties of dental materials, mostly enamel and dentin, to afford them with better adhesion to resins and improved biological properties, such as antimicrobial activity, started more than 20 years ago, but no general overview has been written to our knowledge. (3) Results: the present review is aimed to show that molecules from all the major classes of polyphenolics allow for low coast improvements of dental materials and engineering of dental tissues.

## 1. Introduction

**Figure 1:**
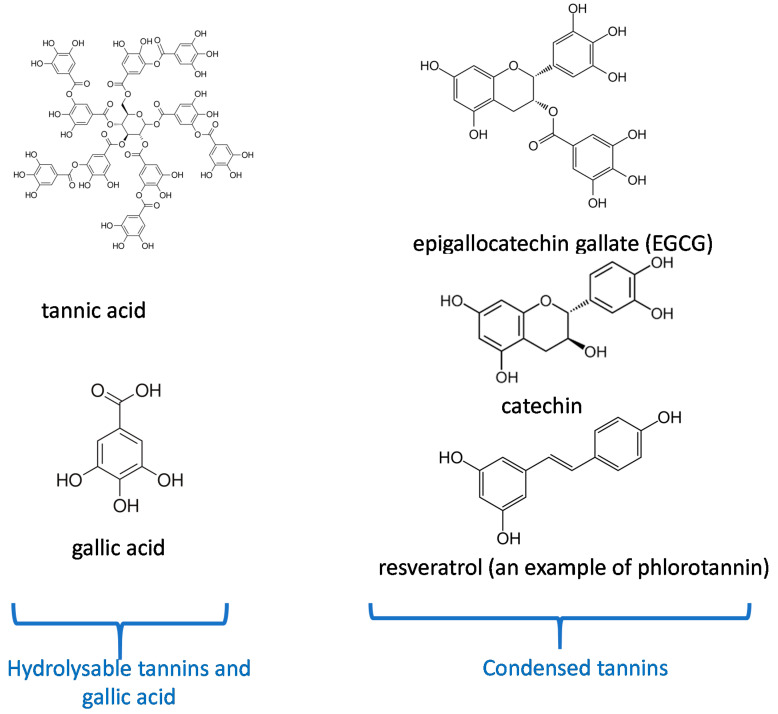
Chemical structure of the most important polyphenols used in dentistry according to their classification [10].

The entry point for nutrients and food in the body is the oral cavity. Tea and some plants contain natural polyphenols [1]. Polyphenols are secondary metabolites defined by the presence of a minimal number of phenol groups [2,3]. Polyphenols play an interesting role in the oral cavity against many diseases, infections, and oral cancers [4]. This is because they have significant features such as antibacterial activities [5], antioxidant effects [6] in the oral cavity. More recently, many investigations have shown that polyphenols can serve as “processing cofactors” to improve mechanical and functional properties of biomaterials [7]. For the same reasons, they are used in many dental applications. In this study, we focus on the most important polyphenols used in dental applications as tannic acid, catechin, resveratol, gallic acid, and epigallocatechin gallate (Figure 1). Our review is aimed to complete the article written by Catapano-Martinez et al., which described the benefit of polyphenols from food for the dental health [8]. Herein we will not only focus on antibacterial applications but also on the use of polyphenols to improve the adhesion between adhesives and dentin or enamel as well as their role in remineralization processes. 

As a starting point, in addition to their structure dependent properties, many polyphenols spontaneously adhere to the surface of almost all known materials [9] allowing to use them in a vast repertoire of materials functionalization strategies. Those possible post-functionalizations rely on the chemical versatility of polyphenols able to undergo acid-base reactions, oxidation processes, chemical reactivity with nucleophiles in the oxidized state, and also chemical coordination with metallic cations [10]. In particular, polyphenols are able to interact with biological and synthetic macromolecules in either a non-covalent or a covalent manner [11]. We build our description of the dental applications of polyphenols based on their well-accepted classification between condensed and hydrolysable polyphenols [10]. Concerning the different subsections describing the application of each class of polyphenols we rely mostly on the anatomy of the teeth and their contact area with saliva concerning their antibacterial activity.

The purpose of this article is to review the literature in which polyphenols were used in dental treatments and engineering. Our literature review relies on PubMed to identify the articles with the following key search sentences: polyphenols from plants and drinking habits in dental caries, polyphenols in enamel, dentin, root canal and, dental pulp treatments, polyphenols in dental adhesives and cements. At purpose, the review is organized with respect to the different applications of polyphenols in the oral cavity and with respect to the big polyphenol families, each one having its chemical and biological specificities [10]. Condensed polyphenols are stable in water but contain a lower fraction of adjacent hydroxyl groups and are often less active for a given chemical interaction than their hydrolysable counterparts, dense in 1,2 diols, but prone to hydrolysis and, hence, fast degradation in water. We will also address the dental applications of polyphenol mixtures. However, it will be apparent that some investigations were devoted to study different kinds of the dental applications, rendering our classification arbitrary in some aspects. We decided to describe the major application of a given investigation in the subsection where the study gave the more pertinent results without neglecting to describe the other applications of the used polyphenol/polyphenol mixture. 

## 2. Condensed Polyphenols

In this section, we will describe the different major dental applications of condensed polyphenols based on flavonoids and on phlorotannins, such as resveratrol, emphasizing on the mechanism of their action as well as on the effective concentration range.

### 2.1. Dentin Modifier, Dentin Pretreatment, Collagen Cross-Linking, and Resin-Dentin Stability

Dentin is the substance located under the enamel layer. It contains essentially hydroxyapatite crystals and a collagen matrix [12,13]. Collagenase and different protease enzymes (Cysteine cathepsins and matrix metalloproteinases) have been reported to cut collagen chains and are responsible for collagen degradation [14,15]. The adhesion of an adhesive-resin material to dentin depends on the formation and the stability of a hybrid zone (zone where the resin infiltrates into the collagen fibrils of the dentin matrix) that forms a micromechanical interlocking between the resin and the dentin matrix [16]. Therefore, the stabilization of collagen in dentin and in the hybrid layer is possible by preventing the hydrolytic and enzymatic degradation catalyzed by the matrix metalloproteinases and the Cysteine cathepsins.

Proanthocyanidins are secondary class of non-hydrolysable plant metabolites used to pretreat dentin, to enhance its mechanical properties and to reduce the collagen digestion. Thus, proanthocyanidins are a clinical agent for dentin bio-modification [17,18]. The monomeric polyphenolic unit in proanthocyanidins interacts with collagen I, essentially with proline residues, to provide a stable interaction between the resin and dentin for 12 months. The stability of this interaction with time is due to a reduced biodegradation of the dentin matrix with low collagen digestion [19]. Tang et al. [20] showed that the use of 15% “*w*/*v*” of grape seed extract (GSE), which contains proanthocyanidins, in contact with demineralized dentin protects the collagen matrix against degradation. They reported that using a solution with 15% of GSE for a 2 min treatment promotes the dentin remineralization rate and forms mostly hydroxyapatite crystals.

A comparison between three polyphenol solutions (at 50 g/L), quercetin, proanthocyanidins and baicalein, showed that the baicalein has the highest effect in the protection of the dentin matrix against collagenase digestion [21].

The formation of polyphenol induced cross-links in the collagen matrix provides cohesion and makes it more resistant to degradation [22].

Liu et al. [23] reported that using 1% “*w*/*w*” of grape seed extract ((+)-catechin, (−)-catechin, (−)-epicatechin, (−)-epigallocatechin, (−)-epicatechin gallate, (−)-epigallocatechin gallate, procyanidin B2, and a pCT-pCT dimer) for 1 min stabilizes the demineralized dentin and provides the cross-linked dentin-collagen complex.

The strong interactions with collagen have necessarily some effect on the mechanical properties of dental tissues: indeed oligomeric proanthocyanidins enhance the elastic modulus of dentin, [24,25]. Concerning the resistance of enamel to dental abrasion was made using mammals’ teeth [26]. Using a hardness test (Vickers indenter), it was shown that saliva containing polyphenol compounds (0.1 M epigallocatechin gallate) in contact with the enamel surfaces showed a greater resistance against abrasion than the enamel put in contact with saliva without added polyphenols.

In addition to the intrinsic mechanical properties of dental tissues, their interaction with other materials, such as resins, needs to be improved. To that aim, dental restorations require tooth surface preparation, this preparation creating a layer of dentinal debris called the “smear layer”. Acid conditioners of dentin are recommended to achieve a clean dentin surface to subsequently provide the required bond strength [27,28,29].

Epigallocatechin-3-gallate (ECGC) solutions with different concentrations (0.02, 0.1, and 0.5% *w*/*v*) were used in dentin pretreatment followed by etch-and-rinse adhesive [30]. The dentin treated with a concentration of 0.5% showed a lower bond strength for resins after one day than dentin treated with solutions at the other concentrations. In contrast, dentin treated with all the polyphenol concentrations preserves its bond strength values for resins during 6 months [30]. Accordingly, Singh et al. [31] reported that dentin treated with ECGC at 0.1% *w*/*v* preserves the bond strength for 6 months in an etch-and-rinse adhesive system. Contrarily, different concentrations of EGCG (0.02, 0.2 and 0.5% *w*/*v*) followed by two-step etch-and-rinse adhesive system had not preserved the bond strength for 1 year of storage in water [32]. These authors reported that the pretreatment with ECGC could reduce the nanoleakage of the resin-dentin interface in time hence reducing the bond strength values. Costa et al. [33] compared the effect of 0.1% of ECGC and 2% *w*/*v* of chlorhexidine as dentin pretreatment to a self-etch system. They demonstrated that the ECGC did not affect the bond strength. The dentin having undergone chlorhexidine pretreatment showed lower values of bond strength after 24 h and 6 months of ageing in water. Another study compared the effect of 0.2 M ECGC and 0.2 M catechin as dentin pretreatment for 1 h in self-etch and rinse-and-etch systems [34]. ECGC treated samples revealed a higher bond strength compared with catechin treated samples due maybe to the higher number of hydroxyl groups in EGCG (Figure 1), thus the ability to establish more hydrogen bond interactions.

ECGC enhances the bond strength of fiber post (a direct restorative dental material) bonded with adhesive-resin and cemented with dual-cure composite resin cement to intraradicular dentin which was treated with sodium hypochlorite. In this study, a push-out test was used to evaluate the bond strength. The authors demonstrated that using an ECGC solution at 400 µg/mL for 1 min as the final irrigation in intraradicular dentin, treated with sodium hypochlorite, increased the push-out strength and bond stability of fiber post for a self-etching and an etch-and-rinse adhesive system [35]. Accordingly, in the study of Pheenithicharoenkul et al. [36], the use of a 1 mg/mL ECGC solution (for 10 min) or ethylenediaminetetraacetic acid (EDTA) at 17% *w*/*v* (for 5 min), followed by ECGC (for 5 min) as the final canal irrigation, demonstrated a higher bond strength than the use of EDTA alone or EDTA with sodium hypochlorite without ECGC in the final canal irrigation solution.

Bonding the dental restoration to the structure of the tooth becomes a routine in dental practice and requires various properties, such as a good sealing, high bonding strength to tooth surfaces, durability over time, low toxicity, and low degradation rate [37,38,39]. 

Epigallocatechin-3-gallate (EGCG) is a polyphenol which has antibacterial and antioxidant activities [40,41] as will be described in Section 2.3. In addition to those properties, EGCG was incorporated in dental adhesive-resins for its inhibitory effects of matrix-metalloproteinases (MMPs) and cysteine cathepsins. Indeed, those proteins are major players in the degradation and perturbation of the resin-dentin interface [42,43]. 

At concentrations of 0.5% and 1% (*w*/*v*), EGCG incorporated in an adhesive resin could increase the bond strength values and the longevity of adhesive-dentin bond for 6–12 months [44,45]. In contrast to the previous investigation, the addition of 0.01 and 0.1% (*w*/*v*) of EGCG to adhesive-resin compounds did not affect the resin-dentin bond, but could reduce the solubility of the adhesive-resin in water [42]. Yu et al. [46] showed that EGCG, in addition to its antibacterial activity, increases the bond strength of root canal sealer to dentin. This result was obtained using a push-out test after a thermocycling procedure (5000 cycles).

The dental adhesive-resin incorporates many toxic compounds such as Bis-GMA, TEGDMA, and Bis-phenol A [47]. Fonseca et al. [43] demonstrated that the presence of 0.5–1% (*w*/*w*) of EGCG in the adhesive-resin could reduce the toxicity, reduce the solubility and the water sorption of this adhesive. These concentrations (0.5–1%) could maintain the hybrid layer and preserve the bond strength over time.

Glass ionomer cements were also blended with 0.1% (*w*/*w*) EGCG to improve their antibacterial activity and to increase their mechanical properties such as the flexural strength and the hardness [48].

Dental adhesive-resins were also modified with quercetin, which is known as an amphiphilic antioxidant. The addition of 500 µg/mL quercetin to dental adhesive could give an antibacterial effect and preserve its bond strength by inhibiting the collagenase activity [49].

Gotti et al. [50] analyzed the effect of the addition of 5% (*w*/*w*) quercetin to a two-step etch-and-rinse, two-step self-etch and one step self-etch adhesive system on bond strength durability to dentin surfaces after two storage periods in water (24 h and 6 months). They demonstrated a negative effect on the bond strength of these adhesives after 24 h. In contrast, for a 6 months storage period, the two-step etch-and-rinse and the two-step self-etch adhesive systems incorporating quercetin increased the bond strength, whilst the one step self-etch adhesive incorporated with quercetin maintained the bond strength durability.

### 2.2. Remineralization, Cell Viability, and Differentiation

ECGC and epicatechin gallate (ECG) (10 mmol/L) did not affect the dental pulp cells viability [51].

Similarly, Lim et al. [52] reported that epicatechin (0.01, 0.05, or 0.1 mM) as a collagen cross-linker did not affect the cell viability and induced a positive effect on the proliferation and differentiation of human dental pulp cells.

Three flavonoids (quercetin, genistein, and baicalin) and phenamil -an osteoblast differentiation molecule- were used to test their cytotoxicity and their osteoblast differentiation activity on dental human cells [53]. The used concentrations, ranging from 1 to 25 µM, did not alter the cell viability. Among the three tested polyphenols, phenamil had the strongest influence on alkaline phosphatase activity. In contrast, this in vitro study demonstrated that quercetin had a superior effect to phenamil in the osteogenic differentiation.

**Figure 2:**
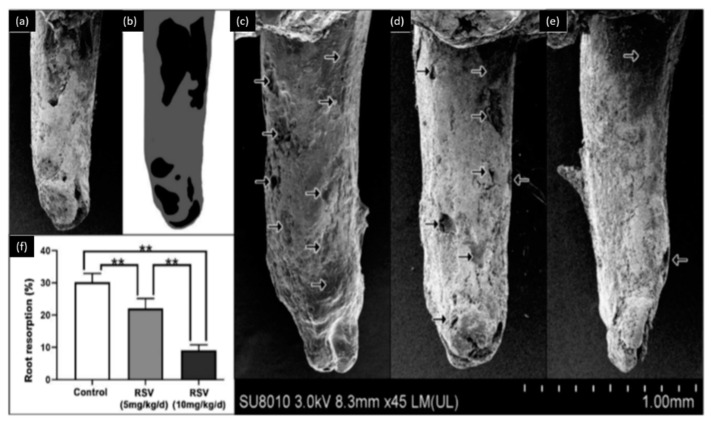
Observation of root resorption. (**a**) SEM image of the tooth, (**b**) root resorption area (black) and the total surface area (grey), (**c**) representative SEM pictures of the control group, (**d**) 5 mg/kg/d resveratrol group, (**e**) 10 mg/kg/d resveratrol group, (**f**) the resorption ratio of the three groups were calculated by dividing the surface of black area by the grey area. (** *p* < 0.01, *n* = 6). Modified from ref. [54] with authorization.

Osteoclasts and osteoblasts play an important role in orthodontic tooth movements. These movements could produce complications, such as root resorption. Liu et al. [54] used two doses of resveratrol (5 mg/kg/day and 10 mg/kg/day) to evaluate its effect on the orthodontic tooth movement and root resorption. This study, performed on rats, demonstrated that resveratrol could inhibit tooth movement during orthodontic treatment and reduce the rate of root resorption during orthodontic therapy as described in (Figure 2). This investigation also showed that resveratrol promotes the osteoblastic activity and reduces the osteoclastic activity during orthodontic therapy. Therefore, resveratrol application during orthodontic therapy could be used as a novel approach to prevent undesired tooth movement (anchorage or relapse).

Tooth development may be perturbed during exposure to high-energy radiations. Barbosa et al. [55] used resveratrol, as a radioprotector, on rats to analyze its effect. Each rat received 100 mg/kg of resveratrol. Barbosa et al. concluded that this dose of resveratrol had no radioprotective effect on the dental tooth structure.

### 2.3. Antibacterial Activity

It has first to be emphasized that the antibacterial activity of polyphenols is related to a combination of mechanisms: implying the inhibition of enzymes implied in the bacterial metabolism as well as the change in the redox balance in the bacterial cell (owing the possible oxidation of catechol groups in quinones) and the reduction in the concentration of metallic cations due to complexation by polyphenolics [10].

Xu et al. [56] showed that a concentration of 31.25 µg/mL of epigallocatechin gallate from green tea inhibits the glucosyltransferase activity of *Streptococcus mutans* and 15.6 µg/mL of the same molecule inhibits 90% of *S. mutans* biofilm formation.

Among condensed polyphenols contained in tea, (−)-epigallocatechin gallate (ECGC) binds to alpha-amylase, which is a salivary enzyme that catalyzes the breakdown of starch. ECGC could inhibit the activity of alpha-amylase by non-competitive inhibition. The antimicrobial activity of EGCG against *Aggregatibacter actinomycetemcomitans* was showed at concentrations higher than 0.5 mg/mL [57].

In the study of Feng et al. [58], the most abundant polyphenols isolated from green tea were catechin and 1,4,6-tri-O-galloyl-βD-glucose. Catechin derivatives as gallocatechin gallate (0.32 mM) and epigallocatechin gallate (0.31 mM) were able to inhibit *S. mutans* glucosyltransferases.

**Figure 3:**
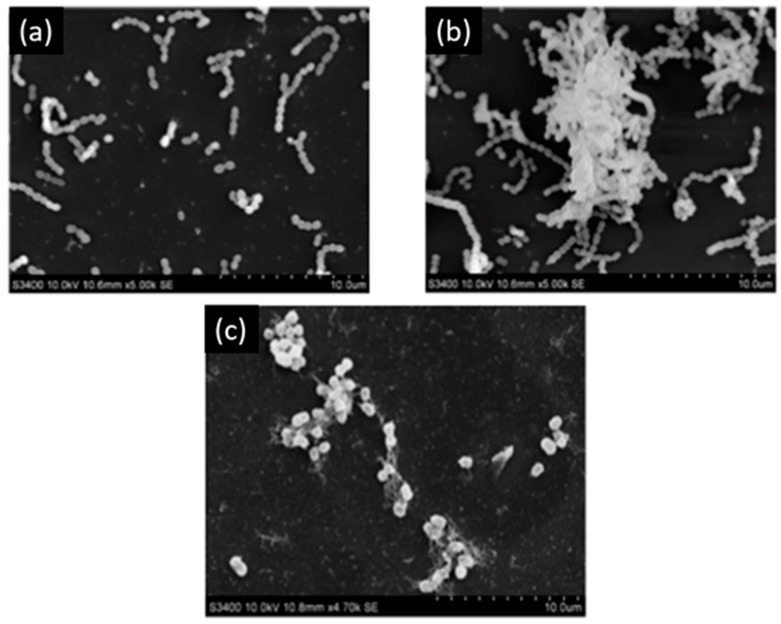
Scanning electron microscopy of *S. mutans*. (**a**) Control; (**b**) untreated *S. mutans* cells after 4 days; (**c**) *S. mutans* cells treated with 250 µg/mL of epigallocatechin-3-gallate-stearate after 4 days. Modified from Ref. [59] with authorization.

Melok et al. [59] showed that the use of 250 µg/mL of epigallocatechin-3-gallate- stearate, which is an esterified derivative of epigallocatechin-3-gallate could completely inhibit *S. mutans* growth and biofilm formation. Using scanning electron microscopy, they evidenced the antibacterial effect of epigallocatechin-3-gallate-stearate after four days of treatment (Figure 3).

ECGC was used as an antibacterial agent against *Enterococcus faecalis* biofilms, which are associated with persistent root canal infections [60]. The minimum inhibitory concentration of ECGC against *E. faecalis* was equal to 5 µg/mL. Lee et al. reported that a concentration of 500 µg/mL applied for 7 days completely eradicated the *E. faecalis* biofilm [60].

EGCG was added to dental restorative composites to afford this material with an antibacterial activity [61]. The addition of 700 µg/mL of EGCG reduces the viability of *S. mutans*. 

To provide some comparative data, Kwon et al. [62] compared the effects of two antibacterial agents and of two cross-linkers (ECGC and glutaraldehyde) on cell viability, odontogenic differentiation, and proliferation of dental pulp cells, and antibacterial activity in collagen scaffolds. The results revealed that the cell viability is reduced in the presence of glutaraldehyde (0.1, 1, 10, and 100 µmol/L) compared with the presence of ECGC (0.1, 1, 10, and 100 µmol/L). In the presence of the highest ECGC concentrations (10–100 µmol/L), the cell viability was nevertheless reduced. It was also concluded that ECGC did not promote the odontogenic differentiation and proliferation by itself but facilitated these processes. The cross-linked collagen produced in the presence of ECGC showed a shorter setting time, a higher compressive strength and a rougher surface. Equal concentrations of ECGC and glutaraldehyde reduced the growth of *S. mutans*.

**Figure 4:**
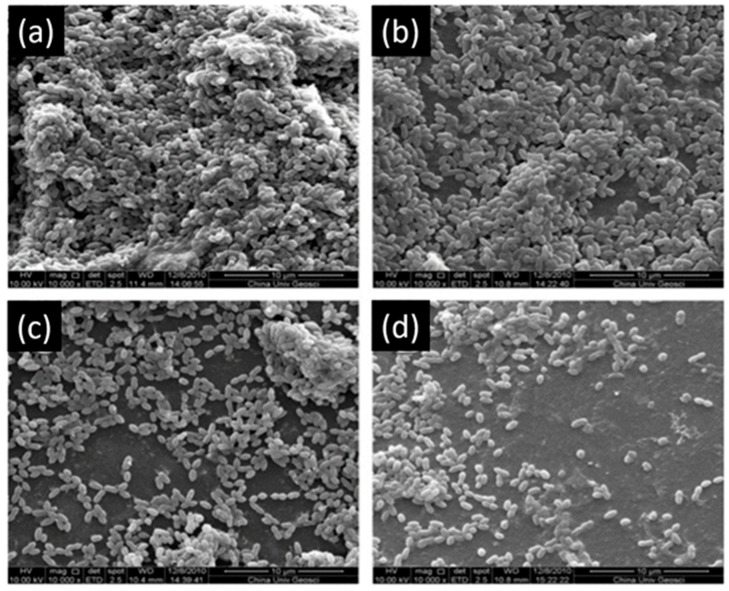
Scanning electron microscopy of *S. mutans* accumulation on specimens. (**a**) Adhesive resin surface without epigallocatechin-3-gallate (ECGC); (**b**) ECGC 100 µg/mL; (**c**) ECGC 200 µg/mL, (**d**) ECGC 300 µg/mL. Biofilms accumulated on (**c**) and (**d**) were not compact after 24 h incubation. Modified from ref. [63] with authorization.

Another study showed that 200 µg/mL and 300 µg/mL of EGCG have an inhibitory effect on the growth of *S. mutans* (Figure 4) and increase the bond strength of the resin to dentin surface. EGCG also preserves the durability of the bond strength for six months [63]. Hence, this investigation is prototypal of the multi-functionality of EGCG in dental applications.

### 2.4. Anti-Inflammatory and Antioxidant Activity

Methacrylate resin-based materials contain triethylene glycol dimethacrylate (TEGDMA), which induces the expression of cyclooxydenase-2. This TEGDMA-induced cyclooxydenase-2 plays a role in dental pulp diseases and pulpit. The cyclooxydenase-2 activity could be suppressed in the presence of 10 and 15 µmol/L of ECGC [64].

Catechins, including epicatechin gallate and epigallocatechin gallate were used to reduce and inhibit the inflammatory factors, which are found in inflamed pulp [65]. In the study of Hirao et al., two catechin concentrations were used (10 and 50 µg/mL) to show that this polyphenol did not affect the viability of the human dental pulp fibroblast cells. Hence, catechins inhibited the effect of interleukins (IL-8 and -6), of monocyte-chemoattractant proteins and of prostaglandins which are receptors stimulating the pro-inflammatory mediators. Contrarily, high concentrations of epigallocatechin gallate (50 µg/mL) did not inhibit the production of prostaglandins in pathogen-associated molecular patterns simulated human dental pulp fibroblasts. Therefore, catechin has an anti-inflammatory effect due to its activity on the inhibition of the cytokines and chemokines in human dental pulp fibroblasts modified with caries-related *S. mutans*, *Streptococcus sanguinis* and *Streptococcus salivarius*, and pathogen-associated molecular patterns. Accordingly, Nakanishi et al. [66] showed the benefits of using epicatechin gallate (ECG) and epigallocatechin gallate (ECGC) to inhibit the expression of pro-inflammatory cytokines and adhesion molecules in human dental pulp cells.

Similarly, EGCG and ECG (dissolved at 20 and 50 ug/mL) reduced the up-regulated expression of vascular endothelial growth factors and cyclooxygenase-2 which are induced pro-inflammatory cytokines in dental pulp cells simulated with lipopolysaccharide (LPS), peptidoglycan (PG), interleukin-1b (IL-1b), or tumor necrosis factor-α (TNF-α) [67].

Wang et al. [51] also demonstrated that the anti-inflammatory activity of ECG and ECGC proceeds through the inhibition of the activity of nuclear factor-kappa B (NF-κB).

Reactive oxygen species (ROS) caused by pulp diseases and dental bleaching agents generate oxidative stress [68]. Park et al. [69] reported the effect of ECGC (5–50 µM) against nitric oxide-induced toxicity of human dental pulp cells which is driven by the ROS production. The Bcl-2 cell family contains anti- and pro-apoptotic proteins, which are important moderators in regulating cell death. In this context, ECGC scavenges the ROS and regulates the expression of the Bcl-2 family preventing the nitric oxide-induced apoptosis.

ROS can also be quenched by butein, which is a plant polyphenol and one of the most active compounds of the *Rhus Verniciflua* plant found in East Asian countries [68]. The study of Lee at al. demonstrated that butein quenches the ROS and suppresses the toxic effects of hydrogen peroxide, which is used as a bleaching agent. The concentrations of butein (2.5–20 µM) had no toxic effect on the dental pulp viability. The maximal heme oxygenase-1 protein expression and heme activity, which exhibit many cytoprotective effects and remove pro-oxidant heme molecules, were attained after 18 h of butein exposure to human dental cells. Nuclear accumulation of nuclear factor-E2 caused by butein treatment increased the promotor activity of antioxidant response elements. Therefore, butein can prevent functional dental cell death and could be used as a protective agent in dental pulp diseases.

Mahmoud Hashemi et al. [70] evaluated the effect of adding 0.5 mg/mL of quercetin to simulated T cells extracted from pulpits with high mobility group box 1 (HMGB1). They demonstrated that quercetin can decrease pro-inflammatory cytokines such as interleukin-6 and -1β with blocking high mobility group box 1 and inhibiting the mitogen activated protein kinase (MAPK) signaling pathway.

Luteolin (used at 35 µmol/L) is also a polyphenol from the flavonol family and was combined with phosphorylated pullulan to decrease the production of inflammatory cytokines [71]. It was reported that the combination of both compounds was less efficient than luteolin alone. This may be due to some strong interactions between both compounds reducing the concentration of available polyphenol.

Concerning resveratrol, a member of the phlorotannin subclass of condensed polyphenols (Figure 1), it was shown to inhibit interleukin (IL-8 and -6) and suppresses the c-Jun N-terminal Kinase (JNK) signaling pathway in dental pulp cells simulated by tumor necrosis factor α (TNFα). TNFα is one of the cytokines that initiates the natural inflammatory response in the dental pulp. In contrast, resveratrol did not inhibit the degradation of IκBα nor the phosphorylation and nuclear translocation of p65 NF-κB in TNFα treatments. The results of this study allowed to hypothesize that resveratrol can be beneficial to decrease pulpal damage during the severe phase of inflammation in vital pulp [72]. Another study reported that using resveratrol at a concentration up to 50 µM had no toxic effect on dental pulp stem cells [73]. Resveratrol raises the activity of Sirtuin 1 (stress-activated nicotinamide adenine dinucleotide-dependent protein deacetylase), which is a mediator of the immune and defense genes in human dental pulp cells [74]. Resveratrol (at 5 µmol/L) activates the function of Sirtiun 1, which can promote the osteogenic differentiation of dental pulp stem cells in inflammation microenvironment through Wnt/β-catenin signal [75].

Resveratrol was added to adhesive-resin materials to promote the biocompatibility of these adhesive-resins, and to reduce the oxidative stress of L929 mouse fibroblast cells without decreasing the bond strength to dentin [76,77].

## 3. Hydrolysable Tannins and Gallic Acid

### 3.1. Dentin Modifier, Dentin Pretreatment, Collagen Cross-Linking, and Resin-Dentin Stability

Four dentin bio-modifiers extracted from different plants were compared such as hydrolysable tannins from Aroeira, condensed tannins from grape seed with cardol and cardanol from cashew nut shell liquid. The results demonstrated that the four groups achieved cross-linking in dentin matrix after 1 min of treatment and the best bio-modifiers were cardol and cardanol [78].

Tannic acid forms stable cross-links with exposed collagen fibrils allowing to increase the resistance against their degradation process [79]. Bedran-Russo et al. [22] reported that solutions containing 10% and 20% (*w*/*v*) of tannic acid could increase the stiffness of demineralized dentin and reduce the enzymatic degradation of collagen most probably due to hydrogen bonds between the biopolymer and tannic acid (TA).

A successful dental root canal treatment depends on various factors such as proper cleaning, and tridimensional filling of the root canal system. The removal of the smear layer and the disinfection of the root canal system is of prime importance during the endodontic therapy [80,81].

Bitter [82] analyzed the effect of using hydrogen peroxide and sodium hypochlorite followed by a solution containing 25% (*w*/*v*) of tannic acid as the final irrigation solution in dental root canal. This study showed that using tannic acid in the final irrigation fluid revealed a smoother and cleaner pulp chamber surface compared with hydrogen peroxide and sodium hypochlorite treatment without tannic acid as final irrigation solution. In addition, Bitter [83] showed that using a 25% (*w*/*v*) solution of tannic acid in contact for 60 s removed the smear layer without broadening the orifice of dentinal tubules, and removed partially the organic material of the dentinal tubules. 

The same group reported that dentin treated with tannic acid had an improved resistance to collagenase degradation [84]. In contrast, another study demonstrated that the application of solutions containing 15, 20, and 25% (*w*/*v*) of tannic acid on dentin surfaces for 5, 10, or 15 min could not totally remove the smear layer owing to the astringent action of tannic acid. They reported that tannic acid itself attached to collagen by means of hydrogen bonds [85]. Different concentrations of tannic acid (2, 5, 10, 15, 20, and 25% (*w*/*v*)) were applied on dentin surfaces for different times (15, 30 and 60 s) [86]. These researchers reported that a low concentration of tannic acid (2 or 5% (*w*/*v*)) applied for 60 s could remove the smear layer, leaving the orifices of dentinal tubules occluded. The dentinal surface that was treated with 20% or 25% (*w*/*v*) of tannic acid for 15s revealed incomplete removal of the smear layer and exposure of some of the dentinal tubules. Accordingly, Bitter [84] tested the permeability of methylene blue in 62 dentinal cavities treated with solutions containing 25% (*w*/*v*) of tannic acid for 15 s. Their results showed that 48 over 62 cavities did not allow for the penetration of methylene blue into the dentinal tubules.

A glass ionomer was blended with 1.5%, 5%, and 10% (*w*/*w*) tannin-fluoride preparation; the 1.5% preparation increased the bond strength of glass ionomer to dentin after one day. The bond strength of glass ionomer modified with tannin-fluoride preparation did not reveal a significant difference after one month [87]. 

Tannic acid incorporated in polycarboxylate cement, as well zinc fluoride, enhances the resistance of dentinal collagen to collagenase and proteolytic enzymes [88].

**Figure 5:**
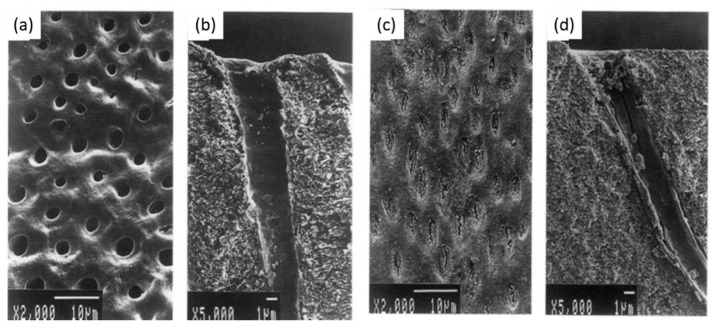
(**a**,**b**) Scanning electron micrographs of dentin hypersensitivity, (**c**,**d**) scanning electron micrographs of dentin treated with fluoride-tannin acid-lanthanum-apatite. Modified from ref. [79] with authorization.

The dentin fluid flow is the main cause of dentin hypersensitivity, which was reduced by using gallic acid/Fe^+3^ complexes (aqueous solutions of FeCl_3_ (1.2 × 10^−3^ M) and gallic acid (0.47 × 10^−3^ M)), which performed for four repeated treatments each lasting over 60 s [89]. This kind of catechol-iron complex is able to deposit on the surface of all known materials [90]. Oh et al. [89] showed that the pyrogallol group of gallic acid binds to dentin and Fe^+3^ ions create stable cross-linked complexes in an aqueous environment. Their scanning electron microscopy observations showed that dentin treated with gallic acid/Fe^+3^ complexes create tight bridge like connections between adjacent peritubular dentin, which resulted in less outward flow. Another complex that is fluoride-tannin acid-lanthanum-apatite was used to reduce the dentinal hypersensitivity [79]. The surfaces of the treated samples were completely covered with fine spherical compounds, and the dentinal tubules were occluded with materials (Figure 5).

Three different antioxidants (solutions containing 10% gallic acid, 10% tannic acid, and 10% ascorbic acid “*w*/*v*”) were used to irrigate the root canal and to evaluate the infiltration of resin sealer in dentinal tubules [91]. The application of gallic acid for 10 min showed the best penetration of resin sealer into dentinal tubules, maybe due to the presence of three vicinal hydroxyl groups in its structure (Figure 1). 

Gallic acid, a polyphenol made from a single aromatic ring (Figure 1) improved its inhibitory effect on matrix metalloproteinases and cysteine cathepsins to improve the durability of bond strength [18]. In contrast, it reduced the mechanical properties of adhesive-resin such as biaxial flexural strength and hardness values [92].

### 3.2. Remineralization, Cell Viability, and Differentiation

The structure of enamel is an essential portion of the tooth, which is exposed in the oral cavity. It is the hardest and most mineralized tissue of the body [93,94]. Salivary pellicles composed of adsorbed macromolecular compounds delivered from saliva, blood, gingival fluids, bacteria, molecules and particles from the diet [95], *Streptococcus mutans (S. mutans)* and other oral bacteria adhere on the surface of enamel. All these substances and particles demineralize this surface resulting in the formation of dental caries [96,97,98]. To reduce the severity of such processes, gallic acid, present in various food and plants can inhibit the enamel demineralization as a calcium chelator and can enhance the remineralization of the demineralized enamel. Thus, gallic acid is a promising agent for enamel remineralization and caries treatments [99,100]. Gallic acid was also used to re-mineralize the early carious enamel, to increase the surface microhardness and simultaneously to reduce the wear resistance of enamel [101]. 

**Figure 6:**
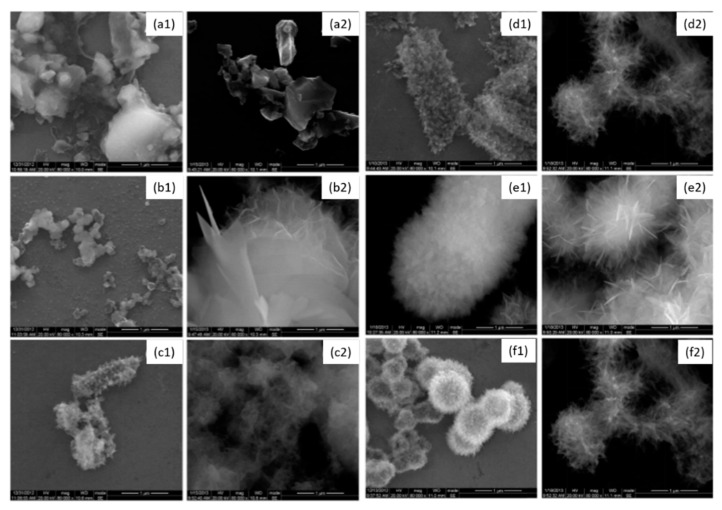
Morphology of hydroxyapatite (**1**) and hydroxyapatite + gallic acid (**2**) crystals at different times; (**a**) = 3 h, (**b**) = 12 h, (**c**) = 24 h, (**d**) = 3 days, (**e**) = 7 days, (**f**) = 14 days at 80,000× magnification. Modified from ref. [102] with authorization.

Enamel is mostly composed of mineral, approximately 96% in weight. Owing to its strong chelation ability with Ca^2+^ cations, gallic acid (GA) was previously used to enhance the remineralization of demineralized enamel [99,100]. Gallic acid (4 g/L) was used to induce the formation of hydroxyapatite (HAP) [102]. It was shown that gallic acid participates in hydroxyapatite formation, limits the crystal growth mainly along the [002] direction and changes the crystal morphology and size. GA-HAP crystals were smaller than the HAP crystals obtained in the absence of polyphenol. The crystal morphology was observed by scanning electron microscopy (Figure 6). It was found that GA-HAP had an urchin-like shape, while loose needle-like crystals were found in HAP formed without additive.

Accordingly, the same authors [103] investigated the morphology and the size changes of crystals as function of the GA concentration (0.05 to 4 g/L). They reported that increasing the concentration in GA reduced the crystal size from 40 to 25 nm. This is the result of gallic acid adsorption on specific crystal faces inhibiting further crystal growth.

### 3.3. Antibacterial Activity

Apacaries gel is a material containing polyphenols from mangosteen extracts and papain as an enzyme [104]. This gel, including polyphenolic compounds, such as gallic acid, which have antibacterial effects, could play an interesting role in the removal of various tissues.

### 3.4. Anti-Inflammatory and Antioxidant Activity

In addition to its potent Ca^2+^ chelator activity, gallic acid is a very active phenolic acid with a high free radical scavenging activity [91].

Surprisingly, it appears that the antibacterial and anti-inflammatory activities of hydrolysable polyphenols were less investigated in the dental field than for their condensed counterparts. This may well be due to their instability in aqueous solutions.

## 4. Polyphenol Mixtures

### 4.1. Dentin Modifier, Dentin Pretreatment, Collagen Cross-Linking, and Resin-Dentin Stability

Quercetin and resveratrol or a mixture of both were used for dentin pretreatment to promote and stabilize the resin-dentin bond [105]. Different concentrations were used (100, 250, 500, or 1000 µg/mL) for 60 s followed by a two-step etch-and-rinse adhesive. The adhesion measurements were made after immersion in water for 1 day and four months, and revealed that the resveratrol and the 1:1 resveratrol-quercetin mixture had the best performance after 4 months. Resveratrol pretreatment showed lower bond strength values than quercetin after 1 day, due probably to its variety of antioxidative mechanisms. Therefore, the polyphenol mixtures used in this study demonstrated a protective effect on the dentin collagen matrix [105].

### 4.2. Remineralization, Cell Viability, and Differentiation

Green tea polyphenols (TP) mixed with nano-sized calcium phosphate particles (TP-CaP) were used in enamel caries lesions [106]. Various TP concentrations (0, 1.2, 12, 18, and 27 mg/mL) were used in the crystal syntheses. The highest concentration (12–27 mg/mL) changed the structure and the crystal size from microsized platelets with porous faces to nano-sized globular particles. The results of this study also showed that adding tea polyphenols to calcium phosphate particles provide an antibacterial effect and enhance the remineralization process. Therefore, this TP-CaP composite could be a promising additive in toothpastes. 

**Figure 7:**
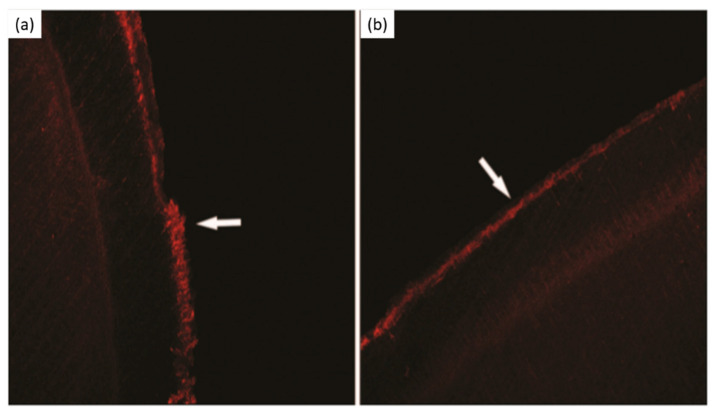
Laser scanning confocal microscopy micrographs. (**a**) Subsurface lesion, (**b**) subsurface lesion after *Galla chinensis* extract treatment. Modified from ref. [107] with authorization.

*Galla chinensis* compounds are polyphenols exhibiting an antioxidant and antibacterial activity [107]. Their study compared the effect of 4000 mg/L of *Galla chinensis* extract with deionized water or a remineralizing solution on the subsurface root lesions and erosive root lesions. They demonstrated that the *Galla chinensis* extract enhances the remineralization of root lesions (Figure 7) more than deionized water or a remineralizing solution containing 1.5 mmol/L CaCl_2_, 0.9 mmol/L KH_2_PO_4_, 130 mmol/L KCl, 1 mmol/L NaN_3_, and 20 mmol/L HEPES buffer. The *Galla chinensis* extract also inhibits the activity of collagenase and protects the collagen fibers against enzymatic degradation [107].

### 4.3. Antibacterial Activity

The oral cavity contains around 750 kinds of bacteria in dental plaques. These bacteria are responsible for dental diseases such as dental caries [108]. Dental caries have been considered the most polymicrobial diseases in the oral cavity [109]. Oral Streptococci, especially *Streptococcus mutans* (*S. mutans*) have been implicated and considered as the main cause and the most cariogenic agent of dental caries in humans [59,96]. These bacteria excrete carbohydrates, such as glucose and sucrose metabolized in organic acids by glucosyltransferases and generate stable biofilms, which affect the mineralized dental surfaces [97,98]. Several studies analyzed the effects of different food components, drinks, and plants containing polyphenol groups in order to prevent the dental decay [109,110]. For instance, tea and cranberry are very rich in polyphenols [111].

Tea is the most popular drink in the world including black, green and oolong teas, which are produced from the plant *Camellia sinensis* [112]. Tea is rich in polyphenols, which have antifungal activities, antibacterial and antioxidant activities, and inhibitory effects on some oral pathogenic microorganisms and oral bacteria such as *S. mutans* [108,113,114,115]. Green tea is an unfermented product mostly containing a mixture of catechins. Fermented tea (black tea) and semi-fermented tea (oolong tea) contain a mixture of catechins, theaflavins, and polymeric thearubigins [112].

The anticariogenic effects of 10% tea polyphenol (10 g of green tea polyphenol extract in 100 mL of dimethyl sulfoxide), 0.05% (w:v) fluoride, 0.2% (w:v) chlorhexidine, and 1:1 solution of 0.2% (*w*/*v*) chlorhexidine and 0.05% (*w*/*v*) fluoride combined were analyzed [116]. This study showed that all the groups had an anticariogenic effect compared with saline, whilst the anticariogenic effect of fluoride-chlorhexidine combined was the highest among those groups.

Ferrazzano et al. [117] compared in an in vivo study the antibacterial effect of tea polyphenol mouthwash (1.6 g of pulverized *Camellia sinensis* leaves was suspended in 40 mL of distilled water at 100 °C for 3 min) and placebo mouthwash against *mutans streptococci* and lactobacilli. Forty milliliters of each mouthwash were used 3 times/day for 7 days. The results showed a significant lowering of the levels of *mutans streptococci* (60%) and a significant lowering of levels of lactobacilli (42.4%) using green tea mouthwash compared with the subjects using placebo mouth-rinse. 

Hambire et al. [118] compared the effect of 0.5% (*w*/*v*) solution of green tea extracted mouthwash with a commercial 0.2% chlorhexidine gluconate mouthwash and 0.05% sodium fluoride mouthwash on the plaque, gingival status, oral hygiene status, and salivary pH. The study was conducted for a period of two weeks on children. The results showed that green tea mouthwash played an important positive role on the criteria tested and no measurable side effects were found with the others mouthwashes tested. Lee et al. [119] showed that chewing green and black tea leaves resulted in higher levels of catechin and theaflavins in the oral cavity than holding tea leaves. Another study demonstrated that tea polyphenols extracted from green and black teas showed, in the 1–10 mM concentration range, a potent (theaflavin) or a moderate (catechin and epicatechin) effect against glucosyltransferase from *S. mutans* [120].

Green tea polyphenols ((+)-catechin (2.9%), (−)-epicatechin (6.8%), (+)-gallocatechin (12.8%), (−)-epigallocatechin (16.5%), (−)-epicatechin gallate (6.6%), (−)-gallocatechin gallate (8.5%), and (−)-epigallocatechin gallate (21.3%) with other compounds such as caffeine (9.9%), sugars (5.1%), amino acids and peptides (2.7%), ash (0.3%), and moisture were added at different concentrations (0.1–0.5% (*w*/*v*)) to diet or water drinking in rats [121]. The results showed that the caries activity was reduced by adding tea polyphenols to the diet. Forty percent of dental caries lesions reduction was observed after a diet containing 0.1% (*w*/*v*) tea polyphenols as well as no toxic effects were observed on rats.

Oolong tea is mostly consumed in China and Taiwan [122]. An in vitro study demonstrated that Oolong tea polyphenols (monomeric and polymeric polyphenols, caffeine, and other components) could be used as antibacterial agents and are able to reduce glucan synthesis by inhibiting the glucosyltransferase activity of *S. mutans* [123]. Accordingly, several studies performed on rats demonstrated that the polymeric polyphenols in oolong tea are the main compounds, which reduce the dental plaque and prevent caries development. The mechanism consists again in the inhibition of glycosyltransferases of *S. mutans* and *Streptococcus sobrinus* [124,125]. The same authors reported that performing mouth rinse with 0.5 mg/mL of oolong tea extracts in 0.2% ethanol solutions, before and after each meal and before sleeping during four days, could reduce the plaque deposition on human teeth [126].

The prevention of dental caries and antibacterial activities of polyphenols in other drinks as cacao, wine, coffee, and barley coffee were studied [127,128]. Barley coffee contains fluoride, zinc ions and phenolic compounds, and has an antibacterial effect on *S. mutans* by changing the hydrophobicity of bacterial cell walls and hence the adhesive capacity of bacteria on hydroxyapatite [129]. Accordingly, Stauder et al. [130] demonstrated the in vitro antibacterial and the in vitro antiadhesive effects of barley coffee against *S. mutans* biofilms and expressed the opinion that the addition of barley coffee compounds to oral health care materials could prevent the *S. mutans*-induced lesions in vivo. They showed that barley coffee contains low molecular mass polyphenols, zinc and fluoride ions and, above all, a high molecular mass melanoidin. After 24 h, barley coffee and the high molecular mass melanoidin fraction had an inhibitory effect (ranging from 82 to 93%), whilst the low molecular mass caused a biofilm reduction of only 36%.

An in vivo study [127] compared the effect of coffee, wine and water consumption on the microbial population of dental supra- and subgingival plaque by the separation of PCR-amplified fragments using the denaturing gradient gel electrophoresis technique. This study showed that the coffee (five cups a day “30 to 40 mL each”) and red wine consumers (couple of glasses a day “200 mL each”) had lower intensity bands observed in supra- and subgingival plaque than water consumers. An in vivo study [114] demonstrated that less biofilms developed on the teeth of consumers drinking higher volumes of wine, tea and/or coffee.

Wine is one of the alcoholic drinks, which is the most frequently consumed by humans. It contains numerous biologically active compounds, which have beneficial effects for the human health [131]. On one hand, several studies showed that wine can alter the tooth color and the long contact duration of this alcoholic beverage with its low pH could promote dental erosion [132,133]. However, on the other hand, red and white wines contain some organic acids and polyphenols; these organic acids such as tartaric, citric, succinic, malic, lactic, and acetic acids could be responsible for the antibacterial activity against *S. mutans*. Conversely, wine polyphenols-catechin and tannin-did not affect the microorganisms [131].

Similarly, wine has strong antibacterial activity against *Porphyromonas gingivalis*, *Aggregatibacter actinomycetemcomitans*, and *Fusobacterium nucleatum*. Hence, the identified active compounds in wine could be used in the prevention and the treatment of periodontal diseases [5]. Resveratrol, flavonols, tannins, and gallic acid are the most represented polyphenols in red wine; these polyphenols and anthocyanins that are present in red wine could have the capacity to inhibit bacterial growth in the oral cavity [134].

Several studies analyzed the antibacterial effect of many polyphenols rich plants, which can be used to prevent dental caries. Hop Bract is a plant that contains a high molecular weight polyphenol, which can inhibit the adherence of the *S. mutans* and 80% of their glucosyltransferase activity at smaller concentrations than the polyphenols extracted from tea [135]. A recent in vivo study demonstrated that the use of 0.1% of Hop Bract polyphenol based mouth rinse (1 min, five times per day) reduces 25.4% of the dental plaque growth over 3 days without teeth discoloration [136]. *Salvadora persica* is a plant containing phenolic, flavonoid, tannins, and alkaloids [137]. The metanolic extracts from this plant contain some polyphenols, such as chrysin-8-c-β-D-glucopyranoside, gallic acid, and ferulic acid. Its total concentration (20 mg/mL) showed high antibacterial activity in vitro against *Staphylococcus aureus* and *Streptococcus* sp. This antibacterial activity was close to or better than the antibacterial activity of ampicillin [138].

*Thymus lamiaceae* comprises more than 200 species. The essential oils of four *Thymus* species contain thymol, and polyphenolic compounds such as phenolic acids, labiate tannins, and flavonoids. They displayed antibacterial activities against *S. mutans.* This study noted that the polyphenols of *Thymus vulgaris* (one of the four species studied) do not have an antibacterial effect but can reduce the initial bacterial colonization on dental enamel in situ [139]. Yamamoto and Ogawa [140] studied the role of *Perilla frutescens* seed that contain various polyphenols such as luteolin, quercetin, gallic acid, and epigallocatechin gallate against different oral bacteria. They observed that the presence of a hydroxyl group at the 3′ position in the flavonoid group of quercetin confers an antibacterial role and prevents the occurrence of dental caries.

The antioxidant and antibacterial effects of the polyphenols compounds contained in *Paullina cupana* (caffeine, epicatechin and catechin) [141], *Oenothera biennis* [142], *Sida urens* L. (Malvaceae) [143], *Cistus incanus* Herbal (flavonols, glycosylated flavonol, catechin, gallic acid) [144], *Ziziphus jujuba* (quercitrin, catechin, gallocatechin) [145], and *Trachyspermum ammi* (Ajwain) [146] showed that these plants could be used to inhibit activities of *S. mutans*; thus, in the prevention of dental caries.

*Candida albicans* is a colonizer of dental caries in children and adults. It has an important role in caries evolution due to the production of some organic acids and dietary sugars in the dental plaque [147]. Polyphenols of green tea (1.25 µg/mL) and Padma Hepaten (0.16 µg/mL) can inhibit the growth of *C. albicans* by 88% and prevent biofilm formation for orthodontic patients [148].

**Figure 8:**
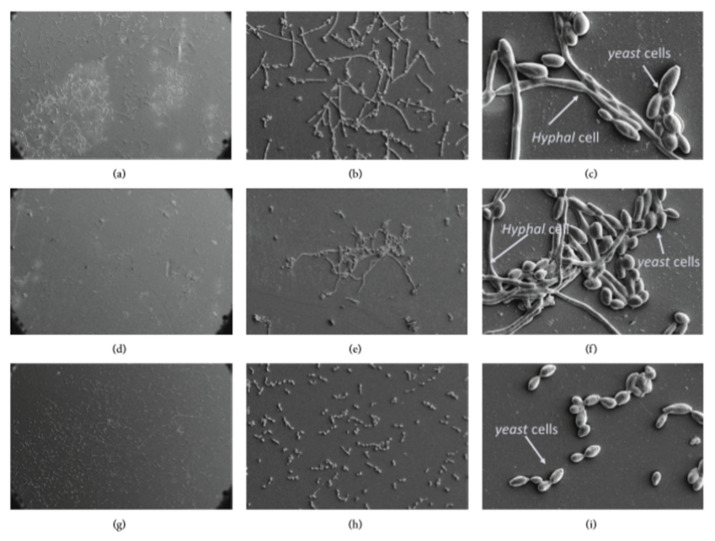
Morphology of the biofilm on orthodontic polyvinyl chloride using scanning electron microscopy at 200×, 1000× and 5000× magnifications, respectively. (**a**–**c**) Untreated group, (**d**–**f**) green tea polyphenols, (**g**–**i**) green tea, and Padma Hepaten polyphenols. Modified from ref. [148] with authorization.

Farkash et al. [148] used scanning electron microscopy to observe the reduction in cell number and the morphology changes of *C. albicans* in the untreated group, in the group treated with green tea polyphenols and in the group treated with green tea polyphenols and Padma Hepaten. The group treated with both plant polyphenols affected *C. albicans* morphology that contains more yeast shaped cells and less hyphal cells (Figure 8).

Accordingly, a recent study from the same group [149] showed the ability of the polyphenols in green tea and Padma Hepaten in the inhibition of the caries-inducing properties of *S. mutans* and *C. albicans*.

*Thymus capitatus* (TC) and *Citrus limon var. pompia* showed antimicrobial capacity against *S. mutans*; but only TC was effective as a fungicidal compound against *C. albicans* able to kill 70% of them. TC is hence a promising plant to prevent caries lesions [150]. 

Polyphenol-rich cranberry could also inhibit the cariogenic factors of *S. mutans*–*C. albicans* biofilms by reducing its acidogenicity and metabolic activity [151].

Poly (methyl methacrylate) (PMMA) is a denture-based material that was blended with Curcumin, a polyphenol with potent biological effects. The results of Alawan et al. showed that 50 µg/mL of curcumin reduces the adhesion of *C. albicans* biofilms on dental materials [152].

Polyphenols contained in tea, such as tannins, catechin, and caffeine, as well as fluoride containing compounds, contribute to increase its resistance against acid solutions [153]. 

Some polyphenols extracted from many plants as Thymus species demonstrated an anti-adherent activity against oral bacteria on the enamel surfaces [139]. An in vitro study on enamel surface using an antioxidant rich-apple (essentially quercetin, epicatechin, procyanidin B2, vitamin C, phloretin, and chlorogenic acid) against *S. mutans* biofilms showed that its polyphenolic compounds decreased the demineralization rate of enamel which is produced by *S. mutans* biofilms [154]. 

A polyphenol containing mouth rinse bath is recommended for its ability to reduce bacterial colonization and adherence on the surface of enamel [155,156,157]. 

### 4.4. Anti-Inflammatory and Antioxidant Activity

Biodegradable chitosan chips containing propolis was used in the treatment of vital pulpotomy [158]. The study of Balata et al. compared the effect of propolis polyphenols (caffeic acid, quercetin, rutin, and chrysin) and formocresol (the gold standard dressing agent in pulpotomy) in the inflammatory response and hard tissue formation. They demonstrated that the propolis samples induced mild or no inflammation compared with formocresol samples. Thus, formulation of propolis extract as chitosan biodegradable chips (3% propolis, 1.8% chitosan, 0.2% hydroxypropyl methylcellulose, 5% propylene glycol) can be used in pulpotomy and in the infected periodontal pockets. These results may be due to the anti-inflammatory activity of propolis, which is rich in polyphenols that inhibit the lipoxygenase pathway of arachidonic acid.

## 5. Summary of the Major Findings Reported in This Review

Taking into account the results discussed in the previous sections, it appears that the different classes of polyphenols have been used in a relatively different manner according to their structure and properties. As apparent from Table 1, summarizing the most relevant studies, -to our modest opinion- condensed polyphenols have found a broad range applications in all the considered fields whereas hydrolysable polyphenols were used preferentially in dentin modification and remineralization, but much less for their antimicrobial and anti-oxidant/inflammatory applications. This may be due to their intrinsic instability being subjected to hydrolysis as well as in the difficulty to obtain them in pure form. Polyphenol mixtures extracted from plants and beverages have been extensively used for their outstanding antimicrobial properties but also in relationship to their availability.

## 6. Future Perspectives and Concluding Remarks

**Figure 9:**
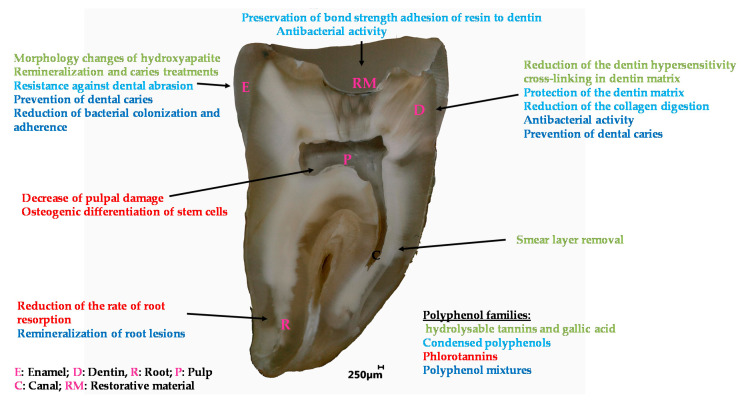
Overview of the applications of polyphenols in the engineering of dental materials.

Polyphenolics whatever their classification as condensed polyphenols, hydrolysable tannins or mixtures thereof allow to easily modify the surface properties of dentin, mainly through interactions with collagen, and enamel affording them with better adhesive properties and antibacterial activity against a broad range of microorganisms present in the proximity of the teeth. All these properties are summarized in Figure 9 and detailed in Table 1.

Future investigations will aim to better understand, and master the use of polyphenols in dental remineralization processes, as they are also able to interact with phosphates.

Even if the literature, describing the use of polyphenols in dental material engineering is vast, as seen in this review, it seems important to develop combinatorial approaches to select the best possible candidates for a required application. The strong interactions between a vast majority of polyphenols and metallic cations, among which Ca^2+^, should also be exploited for the protection and adhesiveness of dental materials. In particular, coatings made from polyphenol metallic cations mixtures [90] may offer some protective applications. The biocompatibility of such metallic–organic hybrid systems has been recently evidenced [159], and highlights the need to investigate them for dental applications.

## Figures and Tables

**Table 1:** Summary of the dental applications of the major classes of polyphenols.

**Condensed Polyphenols**			
**Dentin Modifier, Dentin Pretreatment, Collagen Cross-Linking and Resin-Dentin Stability**			
**Author**	**Polyphenol Used**	**Concentration**	**Effect**
Leme-Kraus [19]	Proanthocyanidins	Enriched grape seed extract, 6.5, 15, 30% (*w*/*v*)	Stable interaction for resin—dentin with low collagen digestion
Santiago [30]	Epigallocatechin-3-gallate	0.02, 0.1% (*w*/*v*)	Preserve bond strength
Singh [31]	Epigallocatechin-3-gallate	0.1% (*w*/*v*)	Preserve bond strength
Kalaiselvam [34]	Epigallocatechin gallate, catechin	0.2 M, 0.2 M, respectively	Epigallocatechin gallate promotes higher bond strength
Yu [35]	Epigallocatechin gallate	400 μg/mL	Increase the bond strength of fiber post
Kwon [62]	Epigallocatechin gallate	0.1, 1, 10, 100 μmol/L	Collagen cross-linked produced in short setting time and high compressive strength
de Macedo [44]	Epigallocatechin gallate	0.5, 0.1% (*w*/*v*)	Increase and preserve the bond strength
Albuquerque [45]	Epigallocatechin gallate	0.5, 0.1% (*w*/*v*)	Increase and preserve the bond strength
Zheng [21]	Flavonols: baicalein, quercetin	50 g/L	Protection of dentin against collagenase digestion
Yang [49]	Flavonols: quercetin	500 μg/mL	Inhibition the collagenase activity
Gotti [50]	Flavonols: quercetin	5% (*w*/*w*)	Preserve the durability of bond strength
Atalayin [77]	Phlorotannins: resveratrol	0.5 μM	Promote biocompatibility of adhesive material without alteration in bond strength
**Remineralization, Cell Viability, and Differentiation**			
Tang [20]	Proanthocyanidins	Enriched grape seed extract, 15% (*w*/*v*)	Protection for the collagen matrix and promote dentin remineralization
Lucas [26]	Epigallocatechin gallate	0.1 M	Enamel resistance against abrasion
Lim [52]	Epicatechin	0.01, 0.05, 0.1 mM	Positive effects on the proliferation of pulp cells
Kim [53]	Flavonols: quercetin, genistein, baicalin	1–25 μM	Osteogenic differentiation
Feng [75]	Phlorotannins: resveratrol	5 μmol/L	Osteogenic differentiation
Liu [54]	Phlorotannins: resveratrol	5 mg/kg/d, 10 mg/kg/d Rat model	Promote osteoblastic activity
**Antibacterial Activity**			
Xu [56]	Epigallocatechin gallate	15.6 μg/mL	Inhibition of *S. mutans* biofilm activity
Hara [57]	Epigallocatechin gallate	More than 0.5 mg/mL	Inhibition of alpha-amylase
Feng [58]	Gallocatechin gallate, epigallocatechin gallate	0.32 mM, 0.31 mM, respectively	Inhibition of *S. mutans* glucosyltransferases
Melok [59]	Epigallocatechin-3-gallate	250 μg/mL	Inhibition of *S. mutans* growth
Lee [60]	Epigallocatechin gallate	500 μg/mL	Eradication of *Enterococcus faecalis* after 7 days
Du [63]	Epigallocatechin gallate	200, 300 μg/mL	Inhibition of *S. mutans* growth and preserve the bond strength of resin-adhesive
**Anti-Inflammatory and Antioxidant Activity**			
Hirao [65]	Catechins	10 and 50 μg/mL	Inhibition of cytokines and chemokines
Nakanishi [66]	Epigallocatechin gallate, epicatechin gallate	20 and 50 μg/mL	Reduce of pro-inflammatory cytokines
Yang [64]	Epigallocatechin gallate	10–15 μmol/L	Suppression of the cyclooxydenase-2
Mahmoud Hashemi [70]	Flavonols: quercetin	0.5 mg/mL	Reduce of pro-inflammatory cytokines
Yonehiro [71]	Flavonols: luteolin	35 μmol/L	Reduce of pro-inflammatory cytokines
Lee [68]	Flavonols: butein	2.5–20 μM	Protective agent in dental pulp diseases
Wang [72]	Phlorotannins: resveratrol	50, 100 μM	Decrease pulpal damage
**Hydrolysable Tannins and Gallic Acid**			
**Dentin Modifier, Dentin Pretreatment, Collagen Cross-Linking, and Resin-Dentin Stability**			
**Author**	**Polyphenol**	**Concentration**	**Effect**
Bedran-Russo [22]	Tannic acid	10%, 20% (*w*/*v*)	Increase dentin stiffness and reduce enzymatic degradation
Bitter [82]	Tannic acid	25% (*w*/*v*)	Smooth and clean pulp chamber
Bitter [83]	Tannic acid	25% (*w*/*v*) for 60s	Remove the smear layer and partially the organic material
Oh [89]	Gallic acid+FeCl_3_	0.47 × 10^−3^ M, 1.2 × 10^−3^ M, respectively	Reduce the dentin hypersensitivity
Mukai [79]	Fluoride-tannin acid-lanthanum-apatite	Containing 5% (*w*/*v*) of tannic acid	Reduce the dentin hypersensitivity
Christopher [91]	Tannic acid, gallic acid	10%, 10%, respectively	Gallic acid shows more infiltration of resin in dental tubules
**Remineralization, Cell Viability, and Differentiation**			
Zhang [99]	Gallic acid	4 g/L	Inhibition of enamel demineralization
Huang [100]	Gallic acid	4 g/L	Inhibition of enamel demineralization
Tang [102,103]	Gallic acid	4 g/L	Change the hydroxyapatite size and morphology
**Polyphenol Mixtures**			
**Dentin Modifier, Dentin Pretreatment, Collagen Cross-Linking, and Resin-Dentin Stability**			
**Author**	**Polyphenol**	**Concentration**	**Effect**
Porto [105]	Quercetin and resveratrol mixture	100–1000 μg/mL	Preserve bond strength and protect dentin matrix
**Remineralization, Cell Viability, and Differentiation**			
Guo [107]	*Galla chinensis*	4000 mg/mL	Enhance the remineralization of root lesions, protect the collagen fibers
**Antibacterial Activity**			
Jazaeri [116]	Tea polyphenol	10% (*w*/*v*)	Anticariogenic effects
Ferrazzano [117]	Tea polyphenol	1.6 g in 40 mL of water	Using as mouthwash reduce *S. mutans*
Hattori [120]	Tea polyphenol	1–10 mM	Reduce the effect of glucosyltransferase of *S. mutans*
Sakanaka [121]	Green tea polyphenols	0.1–0.5% (*w*/*v*) Rat model	Reduce caries activity
Ooshima [126]	Oolong tea extracts	0.5 mg/mL	Reduce the plaque deposition on teeth
Signoretto [127]	Coffee, wine	30–40 mL/d, 400mL/d, respectively	Effective on supra- and subgingival plaque
Shinada [136]	Hop Bract polyphenol	0.1% Mouth rinse (5 times/d)	Reduce 25.4% of the dental plaque
Khalil [138]	*Salvadora Persica*	20 mh/mL	high activity against *S. aureus* and *Streptococcus* sp.
Farkash [148]	Green tea polyphenols and Padma Hepaten	1.25 μg/mL, 0.16 μg/mL, respectively	Inhibition of *C. albicans* growth (88%)
He [106]	Green tea polyphenols mixed with nano-sized calcium phosphate particles	12–27 mg/mL	Antibacterial activity and increase the remineralization process

## References

[1] Pérez-Jiménez J., Neveu V., Vos F., Scalbert A. (2010). Identification of the 100 richest dietary sources of polyphenols: An application of the Phenol-Explorer database. Eur. J. Clin. Nutr..

[2] Handique J.G., Baruah J.B. (2002). Polyphenolic compounds: An overview. React. Funct. Polym..

[3] Reitzer F., Allais M., Ball V., Meyer F. (2018). Polyphenols at interfaces. Adv. Colloid Interface Sci..

[4] Petti S., Scully C. (2009). Polyphenols, oral health and disease: A review. J. Dent..

[5] Sánchez M.C., Ribeiro-Vidal H., Esteban-Fernández A., Bartolomé B., Figuero E., Moreno-Arribas M.V., Sanz M., Herrera D. (2019). Antimicrobial activity of red wine and oenological extracts against periodontal pathogens in a validated oral biofilm model. BMC Complement. Altern. Med..

[6] Fibach E., Ginsburg I. (2015). The Antioxidant Effect of Fermented Papaya Preparation in the Oral Cavity. Phytother. Res..

[7] Shavandi A., Bekhit A.E.-D.A., Saeedi P., Izadifar Z., Bekhit A.A., Khademhosseini A. (2018). Polyphenol uses in biomaterials engineering. Biomaterials.

[8] Catapano-Martinez D., Boyce M., Garland M. (2018). The Protective Role of Polyphenols in Oral Health. Decis. Dent..

[9] Sileika T.S., Barrett D.G., Zhang R., Lau K.H.A., Messersmith P.B. (2013). Colorless multifunctional coatings inspired by polyphenols found in tea, chocolate, and wine. Angew. Chem. Int. Ed. Engl..

[10] Quideau S., Deffieux D., Douat-Casassus C., Pouységu L. (2011). Plant polyphenols: Chemical properties, biological activities, and synthesis. Angew. Chem. Int. Ed. Engl..

[11] Le Bourvellec C., Renard C.M.G.C. (2012). Interactions between polyphenols and macromolecules: Quantification methods and mechanisms. Crit. Rev. Food Sci. Nutr..

[12] Marshall G.W., Marshall S.J., Kinney J.H., Balooch M. (1997). The dentin substrate: Structure and properties related to bonding. J. Dent..

[13] Kharouf N., Rapp G., Mancino D., Hemmerlé J., Haikel Y., Reitzer F. (2019). Effect of etching the coronal dentin with the rubbing technique on the microtensile bond strength of a universal adhesive system. Dent. Med. Probl..

[14] Osorio R., Yamauti M., Osorio E., Ruiz-Requena M.E., Pashley D., Tay F., Toledano M. (2011). Effect of dentin etching and chlorhexidine application on metalloproteinase-mediated collagen degradation. Eur. J. Oral Sci..

[15] Toledano M., Osorio R., Osorio E., Aguilera F.S., Yamauti M., Pashley D.H., Tay F. (2007). Effect of bacterial collagenase on resin-dentin bonds degradation. J. Mater. Sci. Mater. Med..

[16] Mazzoni A., Nascimento F.D., Carrilho M., Tersariol I., Papa V., Tjäderhane L., Di Lenarda R., Tay F.R., Pashley D.H., Breschi L. (2012). MMP activity in the hybrid layer detected with in situ zymography. J. Dent. Res..

[17] Aguiar T.R., Vidal C.M.P., Phansalkar R.S., Todorova I., Napolitano J.G., McAlpine J.B., Chen S.N., Pauli G.F., Bedran-Russo A.K. (2014). Dentin biomodification potential depends on polyphenol source. J. Dent. Res..

[18] Vidal C.M.P., Leme A.A., Aguiar T.R., Phansalkar R., Nam J.-W., Bisson J., McAlpine J.B., Chen S.-N., Pauli G.F., Bedran-Russo A. (2014). Mimicking the hierarchical functions of dentin collagen cross-links with plant derived phenols and phenolic acids. Langmuir.

[19] Leme-Kraus A.A., Aydin B., Vidal C.M.P., Phansalkar R.M., Nam J.W., McAlpine J., Pauli G.F., Chen S., Bedran-Russo A.K. (2017). Biostability of the Proanthocyanidins-Dentin Complex and Adhesion Studies. J. Dent. Res..

[20] Tang C., Fang M., Liu R., Dou Q., Chai Z., Xiao Y., Chen J. (2013). The role of grape seed extract in the remineralization of demineralized dentine: Micromorphological and physical analyses. Arch. Oral Biol..

[21] Zheng K., Wu S., Chen B., Liao W., Li Y. (2014). [Effect of baicalein and quercetin on enzymatic resistance of dentin collagen]. Zhonghua Kou Qiang Yi Xue Za Zhi.

[22] Bedran-Russo A.K.B., Yoo K.J., Ema K.C., Pashley D.H. (2009). Mechanical properties of tannic-acid-treated dentin matrix. J. Dent. Res..

[23] Liu Y., Bai X., Li S., Liu Y., Keightley A., Wang Y. (2015). Molecular weight and galloylation affect grape seed extract constituents’ ability to cross-link dentin collagen in clinically relevant time. Dent. Mater..

[24] Nam J.-W., Phansalkar R.S., Lankin D.C., Bisson J., McAlpine J.B., Leme A.A., Vidal C.M.P., Ramirez B., Niemitz M., Bedran-Russo A. (2015). Subtle Chemical Shifts Explain the NMR Fingerprints of Oligomeric Proanthocyanidins with High Dentin Biomodification Potency. J. Org. Chem..

[25] Phansalkar R.S., Nam J.-W., Chen S.-N., McAlpine J.B., Napolitano J.G., Leme A., Vidal C.M.P., Aguiar T., Bedran-Russo A.K., Pauli G.F. (2015). A galloylated dimeric proanthocyanidin from grape seed exhibits dentin biomodification potential. Fitoterapia.

[26] Lucas P.W., Wagner M., Al-Fadhalah K., Almusallam A.S., Michael S., Thai L.A., Strait D.S., Swain M.V., van Casteren A., Renno W.M. (2016). Dental abrasion as a cutting process. Interface Focus.

[27] Zhang L., Huang L., Xiong Y., Fang M., Chen J.-H., Ferrari M. (2008). Effect of post-space treatment on retention of fiber posts in different root regions using two self-etching systems. Eur. J. Oral Sci..

[28] Kharouf N., Mancino D., Naji-Amrani A., Eid A., Haikel Y., Hemmerle J. (2019). Effectiveness of Etching by Three Acids on the Morphological and Chemical Features of Dentin Tissue. J. Contemp. Dent. Pract..

[29] Gu X.-H., Mao C.-Y., Liang C., Wang H.-M., Kern M. (2009). Does endodontic post space irrigation affect smear layer removal and bonding effectiveness?. Eur. J. Oral Sci..

[30] Santiago S.L., Osorio R., Neri J.R., Carvalho R.M., Toledano M. (2013). Effect of the flavonoid epigallocatechin-3-gallate on resin-dentin bond strength. J. Adhes. Dent..

[31] Singh P., Nagpal R., Singh U.P. (2017). Effect of dentin biomodifiers on the immediate and long-term bond strengths of a simplified etch and rinse adhesive to dentin. Restor. Dent. Endod..

[32] Fialho M.P.N., Hass V., Nogueira R.P., França F.M.G., Turssi C.P., Basting R.T., Amaral F.L.B. (2019). Effect of epigallocatechin-3- gallate solutions on bond durability at the adhesive interface in caries-affected dentin. J. Mech. Behav. Biomed. Mater..

[33] De Costa C.A.G., Passos V.F., Neri J.R., Mendonça J.S., Santiago S.L. (2019). Effect of Metalloproteinase Inhibitors on Bond Strength of a Self-etching Adhesive on Erosively Demineralized Dentin. J. Adhes. Dent..

[34] Kalaiselvam R., Ganesh A., Rajan M., Kandaswamy D. (2018). Evaluation of bioflavonoids on the immediate and delayed microtensile bond strength of self-etch and total-etch adhesive systems to sound dentin. Indian J. Dent. Res..

[35] Yu H.-H., Zhang L., Xu S., Li F., Yu F., Liu Z.-Y., Huang L., Chen J.-H. (2017). Effects of Epigallocatechin-3-gallate (EGCG) on the bond strength of fiber posts to Sodium hypochlorite (NaOCl) treated intraradicular dentin. Sci. Rep..

[36] Pheenithicharoenkul S., Panichuttra A. (2016). Epigallocatechin-3-gallate increased the push out bond strength of an epoxy resin sealer to root dentin. Dent. Mater. J..

[37] Frassetto A., Breschi L., Turco G., Marchesi G., Di Lenarda R., Tay F.R., Pashley D.H., Cadenaro M. (2016). Mechanisms of degradation of the hybrid layer in adhesive dentistry and therapeutic agents to improve bond durability—A literature review. Dent. Mater..

[38] Dutra-Correa M., Leite A.A.B.V., de Cara S.P.H.M., Diniz I.M.A., Marques M.M., Suffredini I.B., Fernandes M.S., Toma S.H., Araki K., Medeiros I.S. (2018). Antibacterial effects and cytotoxicity of an adhesive containing low concentration of silver nanoparticles. J. Dent..

[39] Mishra P., Jaiswal S., Nikhil V., Gupta S., Jha P., Raj S. (2018). Evaluation of marginal sealing ability of self-adhesive flowable composite resin in Class II composite restoration: An in vitro study. J. Conserv. Dent..

[40] Hong J.-Y., Yon J., Lee J.-S., Lee I.-K., Yang C., Kim M.-S., Choi S.-H., Jung U.-W. (2015). Effects of epigallocatechin-3-gallate on the healing of extraction sockets with a periapical lesion: A pilot study in dogs. J. Biomed. Mater. Res. Part B Appl. Biomater..

[41] De Assis J.S., Lima R.A., Marques Lima J.P., Azevedo Rodrigues L.K., Santiago S.L. (2015). Effect of epigallocatechin-3-gallate application for remaining carious dentin disinfection. J. Conserv. Dent..

[42] Neri J.R., Yamauti M., Feitosa V.P., Pires A.P.M., Araújo R.D.S., Santiago S.L. (2014). Physicochemical properties of a methacrylate-based dental adhesive incorporated with epigallocatechin-3-gallate. Braz. Dent. J..

[43] Fonseca B.M., Barcellos D.C., da Silva T.M., Borges A.L.S., das Neves Cavalcanti B., Prakki A., de Oliveira H.P.M., de Paiva Gonçalves S.E. (2019). Mechanical-physicochemical properties and biocompatibility of catechin-incorporated adhesive resins. J. Appl. Oral Sci..

[44] De Macedo F.A.A., Souza N.O., Lemos M.V.S., De-Paula D.M., Santiago S.L., Feitosa V.P. (2019). Dentin bonding and physicochemical properties of adhesives incorporated with epigallocatechin-3-gallate. Odontology.

[45] Albuquerque N., Neri J.R., Lemos M., Yamauti M., de Sousa F., Santiago S.L. (2019). Effect of Polymeric Microparticles Loaded with Catechin on the Physicochemical Properties of an Adhesive System. Oper. Dent..

[46] Yu H.H., Zhang L., Yu F., Zhou H., Shen L.J., Chen J.H. (2017). [Effects of epigallocatechin-3-gallate modification on the bonding stability of an etch-and-rinse adhesive to intraradicular dentin]. Zhonghua Kou Qiang Yi Xue Za Zhi.

[47] Hanks C.T., Strawn S.E., Wataha J.C., Craig R.G. (1991). Cytotoxic effects of resin components on cultured mammalian fibroblasts. J. Dent. Res..

[48] Hu J., Du X., Huang C., Fu D., Ouyang X., Wang Y. (2013). Antibacterial and physical properties of EGCG-containing glass ionomer cements. J. Dent..

[49] Yang H., Li K., Yan H., Liu S., Wang Y., Huang C. (2017). High-performance therapeutic quercetin-doped adhesive for adhesive-dentin interfaces. Sci. Rep..

[50] Gotti V.B., Feitosa V.P., Sauro S., Correr-Sobrinho L., Leal F.B., Stansbury J.W., Correr A.B. (2015). Effect of antioxidants on the dentin interface bond stability of adhesives exposed to hydrolytic degradation. J. Adhes. Dent..

[51] Wang F., Han Y., Xi S., Lu Y. (2020). Catechins reduce inflammation in lipopolysaccharide-stimulated dental pulp cells by inhibiting activation of the NF-κB pathway. Oral Dis..

[52] Lim E., Lim M.-J., Min K.-S., Kwon Y.-S., Hwang Y.-C., Yu M.-K., Hong C.-U., Lee K.-W. (2016). Effects of epicatechin, a crosslinking agent, on human dental pulp cells cultured in collagen scaffolds. J. Appl. Oral Sci..

[53] Kim J.-G., Son K.M., Park H.C., Zhu T., Kwon J.H., Yang H.-C. (2013). Stimulating effects of quercetin and phenamil on differentiation of human dental pulp cells. Eur. J. Oral Sci..

[54] Liu X.-C., Wang X.-X., Zhang L.-N., Yang F., Nie F.-J., Zhang J. (2020). Inhibitory effects of resveratrol on orthodontic tooth movement and associated root resorption in rats. Arch. Oral Biol..

[55] De Rezende Barbosa G.L., Pimenta L.A., de Almeida S.M. (2016). Micro-CT evaluation of the radioprotective effect of resveratrol on the mandibular incisors of irradiated rats. Braz. Oral Res..

[56] Xu X., Zhou X.D., Wu C.D. (2011). The tea catechin epigallocatechin gallate suppresses cariogenic virulence factors of *Streptococcus mutans*. Antimicrob. Agents Chemother..

[57] Hara K., Ohara M., Hayashi I., Hino T., Nishimura R., Iwasaki Y., Ogawa T., Ohyama Y., Sugiyama M., Amano H. (2012). The green tea polyphenol (-)-epigallocatechin gallate precipitates salivary proteins including alpha-amylase: Biochemical implications for oral health. Eur. J. Oral Sci..

[58] Feng L., Yan Q., Zhang B., Tian X., Wang C., Yu Z., Cui J., Guo D., Ma X., James T.D. (2019). Ratiometric fluorescent probe for sensing *Streptococcus mutans* glucosyltransferase, a key factor in the formation of dental caries. Chem. Commun..

[59] Melok A.L., Lee L.H., Mohamed Yussof S.A., Chu T. (2018). Green Tea Polyphenol Epigallocatechin-3-Gallate-Stearate Inhibits the Growth of *Streptococcus mutans*: A Promising New Approach in Caries Prevention. Dent. J..

[60] Lee P., Tan K.S. (2015). Effects of Epigallocatechin gallate against Enterococcus faecalis biofilm and virulence. Arch. Oral Biol..

[61] Mankovskaia A., Lévesque C.M., Prakki A. (2013). Catechin-incorporated dental copolymers inhibit growth of *Streptococcus mutans*. J. Appl. Oral Sci..

[62] Kwon Y.-S., Kim H.-J., Hwang Y.-C., Rosa V., Yu M.-K., Min K.-S. (2017). Effects of Epigallocatechin Gallate, an Antibacterial Cross-linking Agent, on Proliferation and Differentiation of Human Dental Pulp Cells Cultured in Collagen Scaffolds. J. Endod..

[63] Du X., Huang X., Huang C., Wang Y., Zhang Y. (2012). Epigallocatechin-3-gallate (EGCG) enhances the therapeutic activity of a dental adhesive. J. Dent..

[64] Yang W.-H., Deng Y.-T., Kuo M.Y.-P., Liu C.-M., Chang H.-H., Chang J.Z.-C. (2013). Epigallocatechin-3-gallate blocks triethylene glycol dimethacrylate-induced cyclooxygenase-2 expression by suppressing extracellular signal-regulated kinase in human dental pulp and embryonic palatal mesenchymal cells. J. Endod..

[65] Hirao K., Yumoto H., Nakanishi T., Mukai K., Takahashi K., Takegawa D., Matsuo T. (2010). Tea catechins reduce inflammatory reactions via mitogen-activated protein kinase pathways in toll-like receptor 2 ligand-stimulated dental pulp cells. Life Sci..

[66] Nakanishi T., Mukai K., Yumoto H., Hirao K., Hosokawa Y., Matsuo T. (2010). Anti-inflammatory effect of catechin on cultured human dental pulp cells affected by bacteria-derived factors. Eur. J. Oral Sci..

[67] Nakanishi T., Mukai K., Hosokawa Y., Takegawa D., Matsuo T. (2015). Catechins inhibit vascular endothelial growth factor production and cyclooxygenase-2 expression in human dental pulp cells. Int. Endod. J..

[68] Lee D.-S., Li B., Kim K.-S., Jeong G.-S., Kim E.-C., Kim Y.-C. (2013). Butein protects human dental pulp cells from hydrogen peroxide-induced oxidative toxicity via Nrf2 pathway-dependent heme oxygenase-1 expressions. Toxicol In Vitro.

[69] Park S.Y., Jeong Y.J., Kim S.H., Jung J.Y., Kim W.J. (2013). Epigallocatechin gallate protects against nitric oxide-induced apoptosis via scavenging ROS and modulating the Bcl-2 family in human dental pulp cells. J. Toxicol Sci..

[70] Mahmoud Hashemi A., Solahaye Kahnamouii S., Aghajani H., Frozannia K., Pournasrollah A., Sadegh R., Esmaeeli H., Ghadimi Y., Razmpa E. (2018). Quercetin Decreases Th17 Production by Down-Regulation of MAPK- TLR4 Signaling Pathway on T Cells in Dental Pulpitis. J. Dent..

[71] Yonehiro J., Yoshida Y., Yamashita A., Yoshizawa S., Ohta K., Kamata N., Okihara T., Nishimura F. (2013). Flavonol-containing phosphorylated pullulan may attenuate pulp inflammation. Int. Endod. J..

[72] Wang F.-M., Hu Z., Liu X., Feng J.Q., Augsburger R.A., Gutmann J.L., Glickman G.N. (2019). Resveratrol represses tumor necrosis factor α/c-Jun N-terminal kinase signaling via autophagy in human dental pulp stem cells. Arch. Oral Biol..

[73] Geng Y.-W., Zhang Z., Liu M.-Y., Hu W.-P. (2017). Differentiation of human dental pulp stem cells into neuronal by resveratrol. Cell Biol. Int..

[74] Lee S.-I., Min K.-S., Bae W.-J., Lee Y.-M., Lee S.-Y., Lee E.-S., Kim E.-C. (2011). Role of SIRT1 in heat stress- and lipopolysaccharide-induced immune and defense gene expression in human dental pulp cells. J. Endod..

[75] Feng G., Zheng K., Song D., Xu K., Huang D., Zhang Y., Cao P., Shen S., Zhang J., Feng X. (2016). SIRT1 was involved in TNF-α-promoted osteogenic differentiation of human DPSCs through Wnt/β-catenin signal. In Vitro Cell. Dev. Biol. Anim..

[76] Atalayin C., Armagan G., Konyalioglu S., Kemaloglu H., Tezel H., Ergucu Z., Keser A., Dagci T., Onal B. (2015). The protective effect of resveratrol against dentin bonding agents-induced cytotoxicity. Dent. Mater. J..

[77] Atalayin C., Tezel H., Ergucu Z., Unlu N., Armagan G., Dagci T., Kose T. (2019). The improvement of biocompatibility of adhesives: The effects of resveratrol on biocompatibility and dentin micro-tensile bond strengths of self-etch adhesives. Clin. Oral Investig..

[78] Moreira M.A., Souza N.O., Sousa R.S., Freitas D.Q., Lemos M.V., De Paula D.M., Maia F.J.N., Lomonaco D., Mazzetto S.E., Feitosa V.P. (2017). Efficacy of new natural biomodification agents from Anacardiaceae extracts on dentin collagen cross-linking. Dent. Mater..

[79] Mukai Y., Tomiyama K., Okada S., Mukai K., Negishi H., Fujihara T., Teranaka T. (1998). Dentinal tubule occlusion with lanthanum fluoride and powdered apatite glass ceramics in vitro. Dent. Mater. J..

[80] Mancino D., Kharouf N., Hemmerlé J., Haïkel Y. (2019). Microscopic and Chemical Assessments of the Filling Ability in Oval-Shaped Root Canals Using Two Different Carrier-Based Filling Techniques. Eur. J. Dent..

[81] Haapasalo M., Shen Y., Wang Z., Gao Y. (2014). Irrigation in endodontics. Br. Dent. J..

[82] Bitter N.C. (1989). A 25% tannic acid solution as a root canal irrigant cleanser: A scanning electron microscope study. Oral Surg. Oral Med. Oral Pathol..

[83] Bitter N.C. (1989). Tannic acid for smear layer removal: Pilot study with scanning electron microscope. J. Prosthet. Dent..

[84] Bitter N.C. (1990). The effect of 25% tannic acid on prepared dentin: A scanning electron microscope-methylene blue dye study. J. Prosthet. Dent..

[85] Sabbak S.A., Hassanin M.B. (1998). A scanning electron microscopic study of tooth surface changes induced by tannic acid. J. Prosthet. Dent..

[86] Takahashi H., Okamoto Y., Fujinaka S., Shintani H. (1993). A pilot study of exposure of the smear layer to tannic acid solutions. J. Prosthet. Dent..

[87] Yamaga M., Koide T., Hieda T. (1993). Adhesiveness of glass ionomer cement containing tannin-fluoride preparation (HY agent) to dentin—An evaluation of adding various ratios of HY agent and combination with application diammine silver fluoride. Dent. Mater. J..

[88] Okamoto Y., Shintani H., Yamaki M. (1990). A medicated polycarboxylate cement to prevent complications in composite resin therapy. J. Prosthet. Dent..

[89] Oh S., Gu Y., Perinpanayagam H., Yoo Y.-J., Lee Y., Kim R.K., Chang S.W., Lee J., Zhu Q., Kum K.Y. (2018). Dentinal tubule sealing effects of 532-nm diode-pumped solid-state laser, gallic acid/Fe3+ complex, and three commercial dentin desensitizers. Lasers Med. Sci..

[90] Ejima H., Richardson J.J., Liang K., Best J.P., van Koeverden M.P., Such G.K., Cui J., Caruso F. (2013). One-step assembly of coordination complexes for versatile film and particle engineering. Science.

[91] Christopher S.R., Mathai V., Nair R.S., Angelo J.M.C. (2016). The effect of three different antioxidants on the dentinal tubular penetration of Resilon and Real Seal SE on sodium hypochlorite-treated root canal dentin: An in vitro study. J. Conserv. Dent..

[92] Oguz Ahmet B.S., Sayin Ozel G., Mutluay M.M., Tezvergil Mutluay A. (2019). Effect of gallic acid addition on some mechanical properties of self-adhesive resin cements. Braz. Oral Res..

[93] Hu J.C.-C., Chun Y.-H.P., Al Hazzazzi T., Simmer J.P. (2007). Enamel formation and amelogenesis imperfecta. Cells Tissues Organs (Print).

[94] Lacruz R.S., Habelitz S., Wright J.T., Paine M.L. (2017). Dental enamel formation and implications for oral health and diseases. Physiol. Rev..

[95] Hannig M., Hannig C. (2014). The pellicle and erosion. Monogr. Oral Sci..

[96] Babaeekhou L., Ghane M. (2020). Antimicrobial activity of ginger on cariogenic bacteria: Molecular networking and molecular docking analyses. J. Biomol. Struct. Dyn..

[97] Kolahi J., Fazilati M., Kadivar M. (2009). Towards tooth friendly soft drinks. Med. Hypotheses.

[98] Veloz J.J., Alvear M., Salazar L.A. (2019). Antimicrobial and Antibiofilm Activity against *Streptococcus mutans* of Individual and Mixtures of the Main Polyphenolic Compounds Found in Chilean Propolis. BioMed Res. Int..

[99] Zhang J., Huang X., Huang S., Deng M., Xie X., Liu M., Liu H., Zhou X., Li J., Ten Cate J.M. (2015). Changes in composition and enamel demineralization inhibition activities of gallic acid at different pH values. Acta Odontol. Scand..

[100] Huang X.-L., Liu M.-D., Li J.-Y., Zhou X.-D., ten Cate J.M. (2012). Chemical composition of *Galla chinensis* extract and the effect of its main component(s) on the prevention of enamel demineralization in vitro. Int. J. Oral Sci..

[101] Gao S.S., Qian L.M., Huang S.B., Yu H.Y. (2009). Effect of gallic acid on the wear behavior of early carious enamel. Biomed. Mater..

[102] Tang B., Yuan H., Cheng L., Zhou X., Huang X., Li J. (2015). Effects of gallic acid on the morphology and growth of hydroxyapatite crystals. Arch. Oral Biol..

[103] Tang B., Yuan H., Cheng L., Zhou X., Huang X., Li J. (2015). Control of hydroxyapatite crystal growth by gallic acid. Dent. Mater. J..

[104] Juntavee A., Peerapattana J., Ratanathongkam A., Nualkaew N., Chatchiwiwattana S., Treesuwan P. (2014). The Antibacterial Effects of Apacaries Gel on *Streptococcus mutans*: An in vitro Study. Int. J. Clin. Pediatr. Dent..

[105] Porto I.C.C.M., Nascimento T.G., Oliveira J.M.S., Freitas P.H., Haimeur A., França R. (2018). Use of polyphenols as a strategy to prevent bond degradation in the dentin-resin interface. Eur. J. Oral Sci..

[106] He L., Deng D., Zhou X., Cheng L., ten Cate J.M., Li J., Li X., Crielaard W. (2015). Novel tea polyphenol-modified calcium phosphate nanoparticle and its remineralization potential. J. Biomed. Mater. Res. Part B Appl. Biomater..

[107] Guo B., Que K.-H., Yang J., Wang B., Liang Q.-Q., Xie H.-H. (2012). Effect of *Galla chinensis* on the remineralization of two bovine root lesions morphous in vitro. Int. J. Oral Sci..

[108] Li Y., Jiang X., Hao J., Zhang Y., Huang R. (2019). Tea polyphenols: Application in the control of oral microorganism infectious diseases. Arch. Oral Biol..

[109] Cheng L., Li J., He L., Zhou X. (2015). Natural products and caries prevention. Caries Res..

[110] Slobodníková L., Fialová S., Rendeková K., Kováč J., Mučaji P. (2016). Antibiofilm Activity of Plant Polyphenols. Molecules.

[111] Daglia M., Papetti A., Mascherpa D., Grisoli P., Giusto G., Lingström P., Pratten J., Signoretto C., Spratt D.A., Wilson M. (2011). Plant and fungal food components with potential activity on the development of microbial oral diseases. J. Biomed. Biotechnol..

[112] Friedman M. (2007). Overview of antibacterial, antitoxin, antiviral, and antifungal activities of tea flavonoids and teas. Mol. Nutr. Food Res..

[113] Okubo S., Toda M., Hara Y., Shimamura T. (1991). Antifungal and fungicidal activities of tea extract and catechin against Trichophyton. Nippon Saikingaku Zasshi.

[114] Signoretto C., Burlacchini G., Bianchi F., Cavalleri G., Canepari P. (2006). Differences in microbiological composition of saliva and dental plaque in subjects with different drinking habits. New Microbiol..

[115] Ferrazzano G.F., Amato I., Ingenito A., De Natale A., Pollio A. (2009). Anti-cariogenic effects of polyphenols from plant stimulant beverages (cocoa, coffee, tea). Fitoterapia.

[116] Jazaeri M., Pakdek F., Rezaei-Soufi L., Abdolsamadi H., Rafieian N. (2015). Cariostatic effect of green tea in comparison with common anticariogenic agents: An in vitro study. J. Dent. Res. Dent. Clin. Dent. Prospect..

[117] Ferrazzano G.F., Roberto L., Amato I., Cantile T., Sangianantoni G., Ingenito A. (2011). Antimicrobial properties of green tea extract against cariogenic microflora: An in vivo study. J. Med. Food.

[118] Hambire C.U., Jawade R., Patil A., Wani V.R., Kulkarni A.A., Nehete P.B. (2015). Comparing the antiplaque efficacy of 0.5% *Camellia sinensis* extract, 0.05% sodium fluoride, and 0.2% chlorhexidine gluconate mouthwash in children. J. Int. Soc. Prev. Community Dent..

[119] Lee M.-J., Lambert J.D., Prabhu S., Meng X., Lu H., Maliakal P., Ho C.-T., Yang C.S. (2004). Delivery of tea polyphenols to the oral cavity by green tea leaves and black tea extract. Cancer Epidemiol. Biomark. Prev..

[120] Hattori M., Kusumoto I.T., Namba T., Ishigami T., Hara Y. (1990). Effect of tea polyphenols on glucan synthesis by glucosyltransferase from *Streptococcus mutans*. Chem. Pharm. Bull..

[121] Sakanaka S., Shimura N., Aizawa M., Kim M., Yamamoto T. (1992). Preventive Effect of Green Tea Polyphenols against Dental Caries in Conventional Rats. Biosci. Biotechnol. Biochem..

[122] Goenka P., Sarawgi A., Karun V., Nigam A.G., Dutta S., Marwah N. (2013). *Camellia sinensis* (Tea): Implications and role in preventing dental decay. Pharm. Rev..

[123] Matsumoto M., Hamada S., Ooshima T. (2003). Molecular analysis of the inhibitory effects of oolong tea polyphenols on glucan-binding domain of recombinant glucosyltransferases from *Streptococcus mutans* MT8148. FEMS Microbiol. Lett..

[124] Ooshima T., Minami T., Matsumoto M., Fujiwara T., Sobue S., Hamada S. (1998). Comparison of the cariostatic effects between regimens to administer oolong tea polyphenols in SPF rats. Caries Res..

[125] Ooshima T., Minami T., Aono W., Izumitani A., Sobue S., Fujiwara T., Kawabata S., Hamada S. (1993). Oolong tea polyphenols inhibit experimental dental caries in SPF rats infected with *mutans streptococci*. Caries Res..

[126] Ooshima T., Minami T., Aono W., Tamura Y., Hamada S. (1994). Reduction of dental plaque deposition in humans by oolong tea extract. Caries Res..

[127] Signoretto C., Bianchi F., Burlacchini G., Sivieri F., Spratt D., Canepari P. (2010). Drinking habits are associated with changes in the dental plaque microbial community. J. Clin. Microbiol..

[128] Gaur S., Agnihotri R. (2014). Green tea: A novel functional food for the oral health of older adults. Geriatr Gerontol Int.

[129] Papetti A., Pruzzo C., Daglia M., Grisoli P., Bacciaglia A., Repetto B., Dacarro C., Gazzani G. (2007). Effect of barley coffee on the adhesive properties of oral streptococci. J. Agric. Food Chem..

[130] Stauder M., Papetti A., Daglia M., Vezzulli L., Gazzani G., Varaldo P.E., Pruzzo C. (2010). Inhibitory activity by barley coffee components towards *Streptococcus mutans* biofilm. Curr. Microbiol..

[131] Daglia M., Papetti A., Grisoli P., Aceti C., Dacarro C., Gazzani G. (2007). Antibacterial activity of red and white wine against oral streptococci. J. Agric. Food Chem..

[132] Chikte U.M.E., Naidoo S., Kolze T.J., Grobler S.R. (2005). Patterns of tooth surface loss among winemakers. SADJ.

[133] Borges A., Caneppele T., Luz M., Pucci C., Torres C. (2014). Color stability of resin used for caries infiltration after exposure to different staining solutions. Oper. Dent..

[134] Di Lorenzo A., Bloise N., Meneghini S., Sureda A., Tenore G.C., Visai L., Arciola C.R., Daglia M. (2016). Effect of Winemaking on the Composition of Red Wine as a Source of Polyphenols for Anti-Infective Biomaterials. Materials.

[135] Tagashira M., Uchiyama K., Yoshimura T., Shirota M., Uemitsu N. (1997). Inhibition by hop bract polyphenols of cellular adherence and water-insoluble glucan synthesis of *mutans streptococci*. Biosci. Biotechnol. Biochem..

[136] Shinada K., Tagashira M., Watanabe H., Sopapornamorn P., Kanayama A., Kanda T., Ikeda M., Kawaguchi Y. (2007). Hop bract polyphenols reduced three-day dental plaque regrowth. J. Dent. Res..

[137] Kholkhal W., Ilias F., Traore B., Bekkara F.A., Bekhechi C. (2010). *Salvadora persica*: A rich medicinal plant of polyphenols and alkaloids with biological activity. Nat. Prod. Indian J..

[138] Khalil M.A., El-Sabbagh M.S., El Naggar E.B., El-Erian R.H. (2019). Antibacterial activity of *Salvadora persica* against oral pathogenic bacterial isolates. Niger. J. Clin. Pract..

[139] Schött G., Liesegang S., Gaunitz F., Gleß A., Basche S., Hannig C., Speer K. (2017). The chemical composition of the pharmacologically active Thymus species, its antibacterial activity against *Streptococcus mutans* and the antiadherent effects of T. vulgaris on the bacterial colonization of the in situ pellicle. Fitoterapia.

[140] Yamamoto H., Ogawa T. (2002). Antimicrobial activity of perilla seed polyphenols against oral pathogenic bacteria. Biosci. Biotechnol. Biochem..

[141] Yamaguti-Sasaki E., Ito L.A., Canteli V.C.D., Ushirobira T.M.A., Ueda-Nakamura T., Dias Filho B.P., Nakamura C.V., de Mello J.C.P. (2007). Antioxidant capacity and in vitro prevention of dental plaque formation by extracts and condensed tannins of Paullinia cupana. Molecules.

[142] Matsumoto-Nakano M., Nagayama K., Kitagori H., Fujita K., Inagaki S., Takashima Y., Tamesada M., Kawabata S., Ooshima T. (2011). Inhibitory effects of *Oenothera biennis* (evening primrose) seed extract on *Streptococcus mutans* and *S. mutans*-induced dental caries in rats. Caries Res..

[143] Konaté K., Zerbo P., Ouédraogo M., Dibala C.I., Adama H., Sytar O., Brestic M., Barro N. (2013). Anti-nociceptive properties in rodents and the possibility of using polyphenol-rich fractions from *Sida urens* L. (Malvaceae) against of dental caries bacteria. Ann. Clin. Microbiol. Antimicrob..

[144] Wittpahl G., Kölling-Speer I., Basche S., Herrmann E., Hannig M., Speer K., Hannig C. (2015). The Polyphenolic Composition of *Cistus incanus* Herbal Tea and Its Antibacterial and Anti-adherent Activity against *Streptococcus mutans*. Planta Med..

[145] Damiano S., Forino M., De A., Vitali L.A., Lupidi G., Taglialatela-Scafati O. (2017). Antioxidant and antibiofilm activities of secondary metabolites from *Ziziphus jujuba* leaves used for infusion preparation. Food Chem..

[146] Dadpe M.V., Dhore S.V., Dahake P.T., Kale Y.J., Kendre S.B., Siddiqui A.G. (2018). Evaluation of antimicrobial efficacy of *Trachyspermum ammi* (Ajwain) oil and chlorhexidine against oral bacteria: An in vitro study. J. Indian Soc. Pedod. Prev. Dent..

[147] Klinke T., Kneist S., de Soet J.J., Kuhlisch E., Mauersberger S., Forster A., Klimm W. (2009). Acid production by oral strains of *Candida albicans* and lactobacilli. Caries Res..

[148] Farkash Y., Feldman M., Ginsburg I., Steinberg D., Shalish M. (2018). Green Tea Polyphenols and Padma Hepaten Inhibit *Candida albicans* Biofilm Formation. Evid. Based Complement. Altern. Med..

[149] Farkash Y., Feldman M., Ginsburg I., Steinberg D., Shalish M. (2019). Polyphenols Inhibit *Candida albicans* and *Streptococcus mutans* Biofilm Formation. Dent. J..

[150] Pinna R., Filigheddu E., Juliano C., Palmieri A., Manconi M., D’hallewin G., Petretto G., Maioli M., Caddeo C., Manca M.L. (2019). Antimicrobial Effect of Thymuscapitatus and Citruslimon var. pompia as Raw Extracts and Nanovesicles. Pharmaceutics.

[151] Philip N., Leishman S.J., Bandara H., Walsh L.J. (2019). Polyphenol-Rich Cranberry Extracts Modulate Virulence of *Streptococcus mutans*-*Candida albicans* Biofilms Implicated in the Pathogenesis of Early Childhood Caries. Pediatr. Dent..

[152] Alalwan H., Rajendran R., Lappin D.F., Combet E., Shahzad M., Robertson D., Nile C.J., Williams C., Ramage G. (2017). The Anti-Adhesive Effect of Curcumin on *Candida albicans* Biofilms on Denture Materials. Front. Microbiol..

[153] Yu H., Oho T., Xu L.X. (1995). Effects of several tea components on acid resistance of human tooth enamel. J. Dent..

[154] Giacaman R.A., Contzen M.P., Yuri J.A., Muñoz-Sandoval C. (2014). Anticaries effect of an antioxidant-rich apple concentrate on enamel in an experimental biofilm-demineralization model. J. Appl. Microbiol..

[155] Hannig C., Spitzmüller B., Knausenberger S., Hoth-Hannig W., Hellwig E., Hannig M. (2008). Detection and activity of peroxidase in the in situ formed enamel pellicle. Arch. Oral Biol..

[156] Hannig C., Spitzmüller B., Hoth-Hannig W., Hannig M. (2011). Targeted immobilisation of lysozyme in the enamel pellicle from different solutions. Clin. Oral Investig..

[157] Hannig C., Sorg J., Spitzmüller B., Hannig M., Al-Ahmad A. (2009). Polyphenolic beverages reduce initial bacterial adherence to enamel in situ. J. Dent..

[158] Balata G.F., Abdelhady M.I.S., Mahmoud G.M., Matar M.A., Abd El-Latif A.N. (2018). Formulation of Saudi Propolis into Biodegradable Chitosan Chips for Vital Pulpotomy. Curr. Drug Deliv..

[159] Guo J., Suàstegui M., Sakimoto K.K., Moody V.M., Xiao G., Nocera D.G., Joshi N.S. (2018). Light driven fine chemical production in yeast biohybrids. Science.

